# HuR biological function involves RRM3-mediated dimerization and RNA binding by all three RRMs

**DOI:** 10.1093/nar/gky1138

**Published:** 2018-11-10

**Authors:** Marta Pabis, Grzegorz M Popowicz, Ralf Stehle, David Fernández-Ramos, Sam Asami, Lisa Warner, Sofía M García-Mauriño, Andreas Schlundt, María L Martínez-Chantar, Irene Díaz-Moreno, Michael Sattler

**Affiliations:** 1 Institute of Structural Biology, Helmholtz Zentrum München, Neuherberg, Germany; 2 Center for Integrated Protein Science Munich at Chair Biomolecular NMR Spectroscopy, Department Chemie, Technische Universität München, Lichtenbergstr. 4, 85747 Garching, Germany; 3 Max Planck Research Group hosted by the Malopolska Centre of Biotechnology of the Jagiellonian University, Krakow, Poland; 4 CIC bioGUNE, Centro de Investigación Cooperativa en Biociencias. Technology Park of Bizkaia, 48160 Derio, Bizkaia, Spain; 5 Centro de Investigación Biomédica en Red de Enfermedades Hepáticas y Digestivas (CIBERehd), Instituto de Salud Carlos III, Madrid, Spain; 6 Instituto de Investigaciones Químicas (IIQ)—Centro de Investigaciones Científicas Isla de la Cartuja (cicCartuja), Universidad de Sevilla - Consejo Superior de Investigaciones Científicas (CSIC), Avda. Americo Vespucio 49, 41092 Sevilla, Spain

## Abstract

HuR/ELAVL1 is an RNA-binding protein involved in differentiation and stress response that acts primarily by stabilizing messenger RNA (mRNA) targets. HuR comprises three RNA recognition motifs (RRMs) where the structure and RNA binding of RRM3 and of full-length HuR remain poorly understood. Here, we report crystal structures of RRM3 free and bound to cognate RNAs. Our structural, NMR and biochemical data show that RRM3 mediates canonical RNA interactions and reveal molecular details of a dimerization interface localized on the α-helical face of RRM3. NMR and SAXS analyses indicate that the three RRMs in full-length HuR are flexibly connected in the absence of RNA, while they adopt a more compact arrangement when bound to RNA. Based on these data and crystal structures of tandem RRM1,2-RNA and our RRM3-RNA complexes, we present a structural model of RNA recognition involving all three RRM domains of full-length HuR. Mutational analysis demonstrates that RRM3 dimerization and RNA binding is required for functional activity of full-length HuR *in vitro* and to regulate target mRNAs levels in human cells, thus providing a fine-tuning for HuR activity *in vivo*.

## INTRODUCTION

HuR/ELAVL1 (Human antigen R/Embryonic Lethal Abnormal Vision-Like Protein 1) is a ubiquitously expressed RNA-binding protein implicated in several vital processes such as cell proliferation, differentiation or responses to stress and immune stimuli. Not surprisingly, its knockdown in mice is embryonic lethal (1). Although HuR is enriched in the nucleus under physiological conditions, its main function, mRNA stabilization, takes place in the cytoplasm where it can translocate, for example in response to cellular stress (2). The function of HuR is regulated at several levels. First, the amount of HuR in cells is controlled and adjusted at the level of transcription, polyadenylation and mRNA stability (3,4). Second, posttranslational modifications, such as phosphorylation, ubiquitinylation, neddylation and cleavage by caspases further regulate the cellular levels and localization of HuR protein. Finally, HuR binding to target mRNAs is controlled by its phosphorylation, methylation and ubiquitination (5–10).

Despite this extensive cellular control of HuR abundance, localization and function, the protein is upregulated in many cancer types and its expression levels and cytoplasmic localization are correlated with malignancy. The tumorigenic effect of HuR is proposed to result from the stabilization of mRNAs contributing to cancer development through enhanced cell proliferation and survival, proangiogenic properties, evasion from recognition by the immune system and increase of invasive and metastatic potential of cancer cells (11). Thus, the physiological protective and antiapoptotic role exerted upon stress can turn into an aberration facilitating the growth, survival and metastasis of cancer cells.

HuR specifically recognizes adenine and uridine-rich elements (ARE) and uridine-rich sequences in 3′ untranslated regions (3′UTRs) of mRNAs (12,13). Photoactivable-Ribonucleoside-Enhanced Crosslinking and Immunoprecipitation (PAR-CLIP) experiments have also shown a significant association of HuR with intronic regions (14,15). The binding of HuR to its mRNA targets results mainly in their increased stability, but other functional roles of HuR have also been described (12). For example, it has been reported that HuR can reduce mRNA stability, enhance mRNA export to the cytoplasm and modulate translation (16–19). A nuclear function of HuR has also been reported, where it regulates alternative splicing and polyadenylation (3,20). It is interesting to note that HuR targets often contain consecutive copies of HuR recognition motifs (13–15). This topology is in agreement with a proposed mechanism underlying the stabilization of mRNAs by HuR, according to which HuR binding and multimerization on the mRNA prevents the recruitment of other factors, e.g. microRNAs (miRNAs) that would promote mRNA degradation (4,21,22).

Human HuR belongs to the Hu/ELAV family of proteins. The other human Hu/ELAV proteins, HuB, HuC and HuD, are primarily expressed in neurons (23). HuR orthologues are not only found in vertebrates, but also appeared early in evolution—in *Drosophila melanogaster* (ELAV, RBP9 and FNE) and in *Caenorhabditis elegans* (EXC-7). The group of Hu/ELAV proteins show remarkable sequence conservation and all bind to U- and AU-rich sequences. However, they have different cellular localizations (nucleus, cytoplasm or both) and molecular functions (regulation of mRNA splicing, polyadenylation, stability and translation, or their combinations) (24).

**Figure 1.**
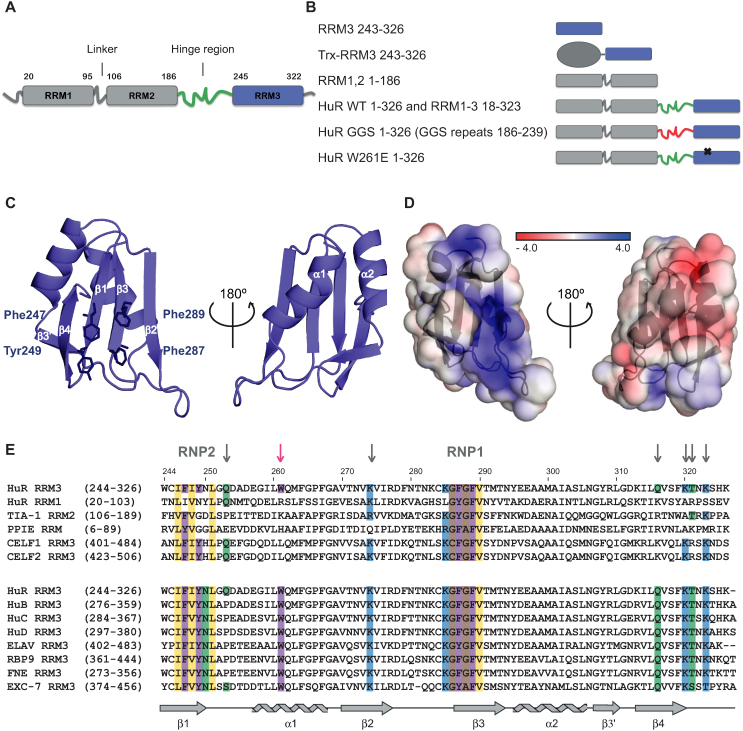
Crystal structure of HuR RRM3. (**A**) Schematic representation of human HuR domain organization. (**B**) List of HuR constructs used in this study with corresponding pictograms. (**C**) Cartoon representation of HuR RRM3 crystal structure. Aromatic amino acids from RNP motifs 1 and 2 are shown as sticks. (**D**) Electrostatic potential surface representation of RRM3. (**E**) Alignment of the human HuR RRM3 amino acid sequence with (i) structurally most similar RRMs and (ii) RRM3 domains of other ELAV and ELAVL proteins from humans (HuB, HuC and HuD), *Drosophila melanogaster* (ELAV, RBP9 and FNE) and *Caenorhabditis elegans* (EXC-7). The sequences were aligned based on primary sequences. Numbers in parentheses indicate the number of the first and last amino acid residue of the selected domain. Numbers above the alignment indicate the amino acid residue number of human HuR RRM3. RNP motives and other residues involved in RNA binding are labelled or indicated with grey arrows. The tryptophan from the dimerization interface is indicated with a pink arrow. Secondary structure elements of HuR RRM3 are shown below the alignments. This panel was generated with the program MView. PDB codes and *Z*-scores of structurally similar RRMs are as follows: TIA-1 RRM2 (3BS9; 10,80), HuR RRM1 (4FXV; 10,44), PPIE (CYP33) RRM (3LPY; 9,32) and CELF2 (ETR3 / CUGBP2) RRM3 (4LJM; 10,65).

Hu/ELAV proteins share a common protein architecture with two consecutive RRMs, separated from the third RRM (RRM3) by a less conserved, flexible hinge region of variable length (25,26). The RRM domain is the most abundant RNA-binding domain. It is composed of two α-helices packed against an antiparallel β-sheet with a β1-α1-β2-β3-α2-β4 topology (27). In HuR, the first two RRMs (RRM1,2) are preceded by a flexible 20 amino acid long N-terminus and connected by a short 12 amino acid linker (Figure 1A). RRM1 is the primary RNA binding domain, but additional contacts between RNA and RRM2, as well as the inter-domain linker strongly improve the RNA binding affinity of RRM1,2 (28). Based on crystal structures and RNA binding data for HuR and HuD, the predicted consensus sequence for RRM1,2 is pyrimidine-rich but only moderately specific: x-U/C-U-x-x-U/C-U-U/C (28,29). The 60-amino acid long hinge region between RRM1,2 and RRM3 encompasses the HuR nucleocytoplasmic shuttling sequence (HNS) and has been implicated in protein-protein interactions (30–32) and/or self-interaction of HuR (21,33). The role of the third RRM (RRM3) is less clear. *In vivo*, two groups reported a distinct effect of overexpression of HuR with a deletion of RRM3 on the stability of a reporter mRNA with *c-fos* ARE (34,35), and it has been suggested that phosphorylation of Ser318 in RRM3 regulates the interaction of full-length HuR with target mRNAs (10,36). *In vitro*, in the context of full-length HuR, both enhancement or a negligible effect of ΔRRM3 on RNA binding have been reported (37,38). RRM3 was described to bind long poly-A, as well as short U- and AU-rich RNAs (39,40). Finally, RRM3 is also implicated in protein-protein interactions and HuR multimerization on mRNA targets (31,37,41). NMR experiments have recently indicated the involvement of the conserved Trp261 in RRM3 dimerization (40). Structural details confirming and explaining how RRM3 performs all of those diverse functions are not available.

Here, we report crystal structures of HuR RRM3 free and bound to U- and AU-rich RNAs. Combined with NMR, SAXS and additional biophysical experiments, we characterize RNA binding and dimerization of RRM3 and full-length HuR. We show that the three HuR RRM domains are dynamic in the absence of RNA but adopt a more compact arrangement upon binding to target RNAs. Full-length HuR with mutations that affect RRM3 RNA binding or dimerization has an impaired function, when overexpressed in colon cancer cells (RKO). Our results reveal an important role of RRM3 in RNA binding and functional activity of HuR.

## MATERIALS AND METHODS

### Plasmids, mutagenesis and synthetic RNAs

The codon optimized sequence of human HuR (NP_001410.2) was obtained from Eurofins and cloned into a series of modified pET-24d vectors with various tags (http://www.helmholtz-muenchen.de/en/pepf/materials/vector-database/bacterial-expression-vectors/index.html). Highest expression and solubility was obtained with pETM44 (N-terminal His_6_ and MBP tag). The following constructs were created based on full-length HuR: sHuR (18–323) in pETM44, RRM1,2 (1–186) in pETM11 (N-terminal His_6_ tag) and RRM3 (243–326) in pET GST-1a (N-terminal His_6_ and GST tag). The tags are cleavable with tobacco etch virus protease (TEV) (pETM11 and pET GST-1a) or human rhinovirus 3C protease (3C) (pETM44). Mutations in HuR and HuR RRM3 were introduced using the Quick Change^®^ Site-Directed Mutagenesis Kit (Stratagene) according to manufacturer's protocol. HuR GGS in pETM11 was created by replacing the linker region between RRM1,2 and RRM3 (186–239) with 18 GGS repeats. Additionally, for crystallization, RRM3 was linked to the C-terminus of *Escherichia coli* thioredoxin 1 (Trx) via a short uncleavable GSAM linker (42).

The HuR-V5 construct (residues 1-326) was cloned into the pEGFPN1 vector along with a C-terminal V5 tag and a stop codon following the tag. HuR-V5 F247/Y249A and W261E were derived from HuR WT by mutagenesis. HuR-V5 ΔRRM3 was subcloned using the In-Fusion technique (Clontech) following the manufacturer's instructions.

PAGE-purified synthetic RNAs were obtained from IBA GmbH. Before use RNAs were resuspended in RNase-free water at 10 mM concentration.

### Protein sample preparation

Unlabeled proteins were expressed in *E. coli* BL21(DE3) in LB medium. The proteins were purified with Ni-NTA agarose (Qiagen) under standard conditions. Tags were cleaved with TEV/3C proteases and removed with a second Ni-NTA purification step. Subsequently, the proteins were purified by size-exclusion chromatography (SEC) on a Superdex™ 75 16/60 prep grade column. HuR WT, its point mutants and sHuR were additionally applied on an ion exchange Resource S column before SEC. ^15^N- and ^15^N-,^13^C-labeled proteins were expressed in minimal (M9) medium supplemented with ^15^NH_4_Cl and ^15^NH_4_Cl, ^13^C-glucose, respectively. For deuterated protein production, the expression was carried out in M9 D_2_O medium supplemented with ^15^NH_4_Cl and ^2^H-glucose.

### NMR spectroscopy

For NMR, protein samples were prepared in 20 mM sodium phosphate buffer (pH 7.0), 200 mM NaCl, 5 mM DTT and 1 mM EDTA. Concentrations of NMR samples of HuR WT, W261E and GGS were in the range of 20 μM (0.7 mg/ml) to 40 μM (1.4 mg ml). For *T*_1ρ_ experiment, the HuR GGS concentration was 150 μM (5.4 mg/ml). The higher concentration was required to obtain sufficient signal-to-noise ratio for the NMR *T*_1ρ_ measurements. For backbone assignment of free RRM3, free RRM1,2 and RRM1,2 bound to AU12 (AUUUUUAUUUUA), the following spectra were collected: HNCA, HNCACB, CBCACONH and ^15^N-edited NOESY. For NMR titrations, RRM3 and RRM1,2 were titrated with increasing amounts of selected RNAs. Chemical shift perturbations were calculated using the following equation: (Δδ(^1^H)^2^ + (0.2 × Δδ(^15^N))^2^)^1/2^. NMR spectra were recorded at 298 K on Bruker 800 and 600 MHz spectrometers equipped with cryoprobes, processed with NMRPipe (43) and analyzed with CCPN analysis. Note, that when superimposed to HSQC spectra TROSY spectra were shifted to match the HSQC signals.

^15^N backbone *T*_1ρ_ relaxation experiments were performed at 800 MHz and 298 K. TROSY detection and temperature compensation were employed, according to (44). The ^15^N rf amplitude for the *T*_1ρ_ spin-lock was set to 2 kHz, while recording an interleaved pseudo-3D, using spin-lock delays of 1, 6, 15, 40 and 100 ms. Exponential decays were fitted per residue to determine *T*_1ρ_ times (*T*_1ρ_ = 1/*R*_1ρ_), setting the experimental error to the standard deviation of the spectral noise. Uncertainties in *T*_1ρ_ were estimated by 1000 Monte Carlo runs.

### Isothermal titration calorimetry

Isothermal titration calorimetry (ITC) measurements were carried out at 298 K using MicroCal ITC200 and PEAQ-ITC calorimeters (Malvern Instruments). Before calorimetry, proteins were dialyzed against 50 mM sodium phosphate buffer (pH 7.0), 200 mM NaCl and 2 mM β-mercaptoethanol. RNAs were injected into the cell containing WT or mutated HuR and RRM3 constructs. After correcting for heat of dilution, the data were fitted to a one-site binding model using the Microcal Origin 7.0 software.

### Crystallization, data collection, structure determination and refinement

For crystallization, HuR RRM3 was expressed with a Trx tag in order to increase its solubility. The protein was concentrated to 6 mg/ml in 20 mM Tris (pH 7.0), 200 mM NaCl, 2 mM β-mercaptoethanol and 6% glycerol. Crystals of the complex were grown at room temperature by vapor diffusion in sitting drops composed of equal volumes (2.25 μl each) of protein solution and crystallization buffer (23% (w/v) PEG 2000 MME, 0.1 M potassium thiocyanate) with addition of 10 mM spermidine tetrahydrochloride (final concentration). For crystallization of RNA-bound HuR RRM3, untagged RRM3 was co-concentrated with U6 (UUUUUU), AU15 (AUUUUUAUUUUAUUU) and AU6tnf (UAUUUA) to a concentration of 6, 3 and 4 mg/ml, respectively. Crystals of RRM3–U6 complex were grown at room temperature by vapor diffusion in sitting drops composed of equal volumes (1 μl each) of protein solution and crystallization buffer (0.1 M Tris pH 8.5 and 2.25 M ammonium sulfate). Crystals of the RRM3–AU15 complex were grown at room temperature by vapor diffusion in sitting drops composed of equal volumes (200 nl each) of protein solution and crystallization buffer (0.1 M HEPES pH 7.5, 10% (w/v) PEG 8000). Crystals of RRM3–AU6tnf complex were grown at room temperature by vapor diffusion in sitting drops composed of equal volumes (200 nL each) of protein solution and crystallization buffer (0.2 M ammonium acetate, 0.01 M calcium chloride, 0.05 M sodium cacodylate pH 6.5 and 10% (w/v) PEG 4000). They were cryoprotected by serial transfer into reservoir solution containing 20% (v/v) glycerol. Cryogenic data were recorded at beamline ID23 of the European Synchrotron Radiation Facility (ESRF) (Trx–RRM3, RRM3–AU15 complex and RRM3-AU6tnf complex) and beamline X06DA at Swiss Light Source (RRM3–U6 complex) (for data collection details see Table 1). The structure of RRM3-Trx was determined by molecular replacement with PHASER using the structure of thioredoxin (2TRX). For RNA-bound RRM3, the structures of the complex were determined using the refined model of free RRM3. The structures were refined in alternating cycles of model correction using COOT and REFMAC5 refinement. Structural quality was checked with PROCHECK. Structural visualization was done with PyMOL (http://pymol.sourceforge.net/). For structure refinement statistics see Table 1.

**Table 1.** Structural statistics of the crystal structures of HuR RRM3 free and bound to RNA

Data collection	RRM3-Trx PDB ID: 6GD1	RRM3 + AU6tnf PDB ID: 6GD3	RRM3 + AU15 PDB ID: 6GD2	RRM3 + U6 PDB ID: 6G2K
Space group	*P* 1 2_1_ 1	*P* 1 2_1_ 1	*P* 1 2_1_ 1	*P* 1 2_1_ 1
**Cell dimensions**				
***a, b, c*** (**Å**)	42.92, 67.66, 70.14	33.74, 79.93, 54.87	34.12, 80.48, 54.44	34.31, 79.74, 51.07
**α, β, γ** (**°**)	90.0, 91.0, 90.0	90.0, 90.6, 90.0	90.0, 90.7, 90.0	90.0, 93.1, 90.0
**Resolution** (**Å**)	8.99–2.01 (2.06–2.01)	6.04–1.35 (1.39–1.35)	8.50–1.90 (1.95–1.90)	8.99–2.01 (2.06–2.01)
***R*_merge_** (**%**)	10.0 (85.2)	7.2 (67.4)	11.6 (60.7)	13.7 (96.5)
**I/σI**	12.48 (2.20)	10.84 (2.18)	9.09 (2.38)	13.40 (2.66)
**Completeness** (**%**)	99.7 (99.4)	98.7 (97.5)	99.6 (100.0)	99.7 (99.6)
**Redundancy**	6.73 (6.32)	3.36 (3.19)	4.06 (3.87)	6.82 (6.73)
**Refinement**				
**Resolution** (**Å**)	8.98–2.01	6.04–1.35	8.50–1.90	8.98–2.01
**No. reflections**	25205	59206	21702	17217
***R*_work_/*R*_free_**	0.1762/0.2442	0.1645/0.1886	0.1916/0.2451	0.1689/0.2349
**No. atoms**				
**Protein**	2923	2003	1956	1890
**Ligand**	–	124	142	120
**Water**	286	297	230	177
***B*-factors** (**Å**)				
**Protein**	40.39	22.40	27.25	29.05
**Ligand**	–	34.28	38.95	36.16
**Water**	48.02	35.88	37.42	41.62
**R.m.s. deviations**				
**Bond lengths** (**Å**)	0.017	0.027	0.017	0.017
**Bond angles** (**°**)	1.819	2.330	1.823	1.767
**Ramachandran plot** (**%**)				
**Most favored**	93.8	95.8	95.2	94.7
**Allowed**	6.3	4.2	4.8	5.3
**Outlier**	0.0	0.0	0.0	0.0

### SAXS data collection and analysis

SAXS measurements were performed on a Rigaku BioSAXS1000 instrument attached to a Rigaku HF007 microfocus rotating anode with a copper target (40 kV, 30 mA) and at the beamline BM29 at ESRF Grenoble with a SEC SAXS setup. The BioSAXS1000 measurements were *q* calibrated with silver behenate. Samples were measured in 8900 s frames checked for beam damage, circular averaged and solvent subtracted by the SAXSLab software (v3.0.2). At minimum, three concentrations were measured from each sample and normalized to concentration to exclude concentration-dependent effects. Pair distance distributions, low resolution models, rigid body models, and ensembles were calculated with the ATSAS package v2.7.0.1 (45). Molecular weights were calculated from the Porod volume.

### Stable RKO cell lines expressing V5-tagged HuR WT, ΔRRM3, F247/Y249A and W261E

RKO colon carcinoma cells were purchased from the American Type Culture Collection (ATCC). Cells were cultured in DMEM supplemented with 10% FBS and 1% penicillin-streptomycin-glutamine at 37°C in a humidified atmosphere of 5% CO_2_–95% air.

RKO cells were transfected with HuR-V5 WT, HuR-V5 ΔRRM3, HuR-V5 F247/Y249A and HuR-V5 W261E plasmids by using Lipofectamine 2000 (Invitrogen), and stably transfected pools of cells were selected with Geneticin (G418) (Gibco).

### Western blot

Cells were lysed in buffer (50 mM Tris pH 8.5, 150 mM NaCl, 5 mM EDTA, 1% NP40, 1 mM complete protease inhibitor cocktail and 50 mM NaF) and centrifuged (10 000 g, 20 min, 4°C). Protein concentration was determined by using the BCA Protein Assay Kit (Thermo Scientific). After quantification, 10 μg of protein were separated by electrophoresis on sodium dodecyl sulfate-polyacrylamide gels and transferred onto membranes. Membranes were blocked with 5% nonfat dry milk in TBS pH 8.0 containing 0.1% Tween-20 (TBST-0.1%), for 1 h at room temperature (RT), washed three times with TBST-0.1% and incubated overnight at 4°C with V5 antibody (1:1000, Invitrogen) and α-tubulin antibody (1:5000 Sigma-Aldrich). Membranes were then washed three times with TBST-0.1% and incubated for 1 h at RT in blocking solution containing secondary anti-mouse antibody conjugated to horseradish peroxidase (Cell Signaling Technology). Immunoreactive proteins were detected by Western Lightning Enhanced Chemiluminescence reagent (ECL, Perkin Elmer) and exposed to X-ray films (Amersham) in a Curix 60 Developer (AGFA).

### Cell cycle analysis

Cell cycle distribution was determined by measuring the cellular DNA content using flow cytometry. In brief, the cells were synchronized in G0 phase by serum deprivation for 16 h and then were released from growth arrest by re-exposure to 10% fetal bovine serum for 6 h, collected by trypsinization and washed with PBS. The collected cells were fixed in 70% ethanol. After the incubation with 10 mg/ml RNase A for 15 min at RT, the cells were resuspended in 0.5 ml 10 μg/ml propidium iodide solution (PI) for staining. The stained cells were monitored by a FACSCanto cytometer (Becton Dickinson). The percentage of cells in the G0/G1, and G2/M phases of the cell cycle was determined using the software FlowJo.

### Ribonucleoprotein immunoprecipitation (RNP-IP) assay

RNA-protein complexes were immunoprecipitated as described before (Fan, Ishmael *et al.*, 2011). The protein lysates for the RNP-IPs were obtained from stably expressing RKO cell lines after overnight serum starvation and 6 h serum re-addition. Cells were washed twice with 1× PBS and lysed in buffer containing 100 mM KCl, 5 mM MgCl_2_, 10 mM HEPES pH 7.0, 0.5% NP-40, 1 mM DTT, RNaseOUT (100 U/ml) and Complete protease inhibitor cocktail (Roche). Homogenates were centrifuged 30 min at 14000 rpm, 4°C, and the supernatant was used for IP of RNA–protein complexes. Fresh whole-cell lysate (150 μg) was first precleaned with 15 μg of IgG2 control (BD Pharmingen) and 25 μl of Protein G-Sepharose beads (Sigma-Aldrich) for 30 min, 4°C with agitation. After spin centrifugation, the supernatant was incubated (1 h, 4°C) with 1 ml of a 50% (v/v) suspension of Protein G-Sepharose beads previously precoated with 30 μg of either IgG2 (BD Pharmingen) or V5 (Invitrogen) antibodies, and washed twice using NT2 buffer (50 mM Tris–HCl pH 7.4, 150 mM NaCl, 1 mM MgCl_2_ and 0.05% NP-40). After incubation, the beads were washed four times (5000 g, 5 min) with 1 ml of ice-cold NT-2 buffer. After the last wash, beads were incubated with 100 μl NT2 buffer containing 20 U DNase I (RNase-free) (Ambion) for 15 min at 37°C, washed with NT2 buffer, and further incubated in 100 μl of NT2 buffer containing 0.1% SDS and 0.5 mg/ml Proteinase K (Roche) for 15 min at 55°C for the isolation of RNA from the immunoprecipitated material. Following centrifugation, the supernatant was collected. RNA from this supernatant was extracted with acid–phenol–CHCl_3_ and precipitated overnight in the presence of 5 μl glycoblue (Ambion), 25 μl sodium acetate pH 5.2 and 625 μl 100% ethanol. Next day, the precipitated RNA was collected by centrifugation, the pellet washed with 70% ethanol, air dried and resuspended in 20 μl of RNase free water (Sigma-Aldrich). Finally, the RNA was analyzed by real time PCR as described below.

### RNA isolation and real-time PCR (qPCR)

RNA was isolated with Trizol (Invitrogen) and its concentration and integrity were determined. PCRs were performed using Applied Biosystems ViiA7 thermocycler by the ΔΔCt method, using GAPDH as reference gene.

## RESULTS

### Structure of RRM3 and RRM3-RNA complexes

In order to investigate the role of RRM3 in HuR function, we set out to optimize its expression and purification conditions to obtain the domain in amounts and purity suitable for biochemical and structural studies. High expression of soluble RRM3 was obtained as fusion with an N-terminal His-GST tag, but tag cleavage lowered the solubility of the protein. To overcome this limitation, we produced RRM3 fused to an N-terminal thioredoxin 1 (Trx) tag via a short uncleavable Gly-Ser-Ala-Met linker (Figure 1B) to improve solubility and suitability for crystallization as described previously (42). The fusion protein (Trx-RRM3) yielded crystals that diffracted to 2.0 Å (see Table 1 for data collection and refinement statistics, Supporting Information; Supplementary Figure S1). As expected, HuR RRM3 adopts a classical RRM fold with a canonical β1-α1-β2-β3-α2-β4 topology (Figure 1C). Similar to a subset of RRMs (e.g. PPIE RRM, HuR RRM1) RRM3 has an additional four amino acid β3′ strand between α2 and β4. The β-sheet surface is predominantly positively charged, providing a suitable interface for the binding of RNA (Figure 1D). The RRM3 RNP motifs (RNP1: IFIYNL, RNP2: KGFGFV) fit the consensus sequence (Figure 1E) suggesting the possibility for RNA binding by stacking of aromatic residues with the base moieties of RNA ligands. A search for structural homologues using PDBeFold (46) yielded TIA-1 RRM2, HuR RRM1, PPIE (CYP33) RRM and CELF2 (ETR3 / CUGBP2) RRM3 as most structurally similar domains (Figure 1E). With the exception of PPIE, these are canonical RNA-binding RRMs (28,47,48). PPIE is distinct in that it can interact with both RNA and proteins (49). The structural similarity to canonical RRMs and the conservation of RNP motifs is indicative of a role of HuR RRM3 in RNA binding.

To characterize RNA binding by RRM3, we performed isothermal titration calorimetry (ITC) using untagged RRM3. We initially selected two 6-mer RNA substrates: a sequence comprising the AUUUA pentamer derived from an ARE in TNF-α 3′UTR, (AU6tnf – UAUUUA), and a poly-U RNA that can be found in class III ARE elements without AUUUA repeats (U6 – UUUUUU). Both RNAs were bound by RRM3, with a *K*_d_ of 156 μM and 19 μM, respectively (Table 2; Supplementary Figure S2A). The higher affinity of RRM3 for the U6 ligand could be due to a preference for U-rich sequences and/or represent an apparent increase in affinity due to multiple binding registers, as has been proposed previously for HuR RRM1,2 (38) and other RNA binding proteins (50). Based on the binding affinities determined by ITC for the binding of RRM3 to UUUUUU (U6), UUUUU (U5) and UUUU (U4), we calculated that the minimal binding site is composed of five nucleotides (Supporting Information, Supplementary Figure S2B and C). It thus seems plausible that the lower *K*_d_ of RRM3 binding to U6 results from both avidity due to binding in multiple registers and preference for U-rich over AUUUA motifs.

**Table 2.** Isothermal titration calorimetry data for RNA binding to HuR and RRM3

Protein	RNA	Sequence	*K*_d_	*n*
RRM3	U6	UUUUUU	19.3 μM ± 0.3	0.8
RRM3	AU6tnf	UAUUUA	155.8 μM ± 32.8	–
RRM3	AU6	UUUAUU	47 μM ± 3.6	0.8
RRM3	GU6	UUUGUU	100.5 μM ± 18.2	1.1
RRM3	GC control	GAGCAC	No binding (NMR)	
RRM3 F247A/Y249A	U6	UUUUUU	No binding	
RRM3 F287A F289A	Not tested (insoluble protein)			
RRM3 F279A	U6	UUUUUU	44.6 μM ± 3.5	0.6
HuR WT	AU17	AUUUUUAUUUUAUUUUU	40.0 nM ± 8.2	0.7
HuR F247A Y249A	AU17	AUUUUUAUUUUAUUUUU	350.9 nM ± 19.9	0.8
HuR F287A F289A	AU17	AUUUUUAUUUUAUUUUU	194.2 nM ± 21.5	0.7
HuR F279A	AU17	AUUUUUAUUUUAUUUUU	28.8 nM ± 4.4	1.0
HuR W261E	AU17	AUUUUUAUUUUAUUUUU	41.0 nM ± 4.8	0.9
HuR GGS	AU17	AUUUUUAUUUUAUUUUU	33.6 nM ± 2.7	0.9
RRM1,2	AU17	AUUUUUAUUUUAUUUUU	214.6 nM ± 14.7	0.9

**Figure 2.**
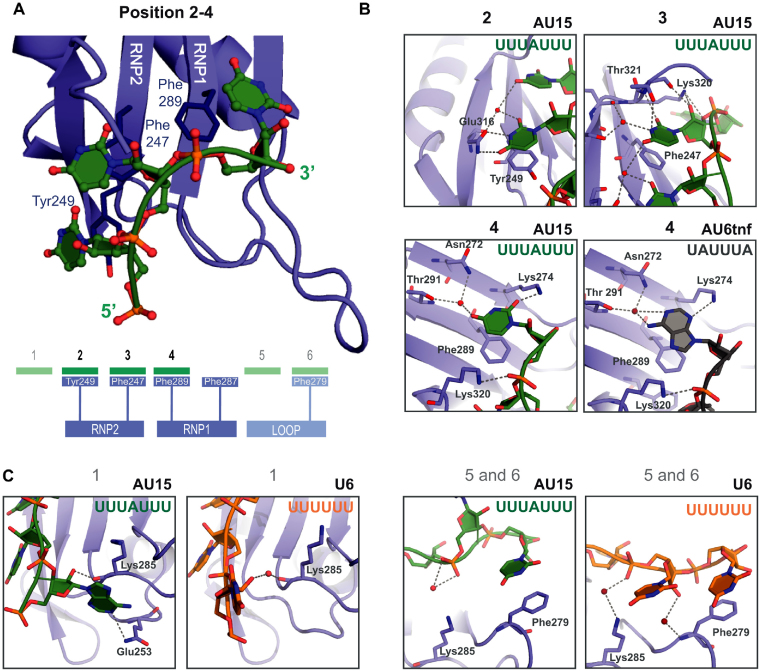
Crystal structure of HuR RRM3 bound to RNA. (**A**) Cartoon representation of the RRM3**-**RNA complex (core interaction) from the RRM3–AU15 crystal. The RNA is shown as a ball-and-stick model with filled rings colored according to atom type (oxygen—red, nitrogen—blue, phosphorus—orange). RNP amino acids involved in stacking interactions with the RNA are shown as sticks. The diagram depicts RNA nucleotides as green rectangles and corresponding RNA binding areas of RRM3 in blue (with aromatic residues presented as rectangles). Darker colors represent the core interactions, while the peripheral ones are presented in lighter colors. (B, C) Zoomed views showing details of RRM3 interaction with RNA. RRM3 is shown as blue cartoon, residues that exhibit contacts with the RNA are depicted as sticks and labeled. RNA ligands are shown in different colors depending on their sequence (AU15—green, U6—orange, AU6tnf—grey) and are additionally colored according to atom type as in (A). Water molecules are shown as red spheres Hydrogen bonds between RRM3 and the RNA (also water-mediated) are indicated as grey dashed lines. (**B**) The binding in position 2 and 3 is identical for all three ligands (U6, AU15, AU6tnf). For position 4, the interaction with uridine (in AU15 RNA, left) and adenine (in AU6tnf RNA, right) are shown. (**C**) Peripheral interactions (positions 1, 4 and 5) that vary depending on the RNA ligand and distinct RRM3 protomers in the asymmetric unit are shown with the same color code as in (B).

We next wished to determine the structural basis for RNA recognition by RRM3. We observed a substantial increase in RRM solubility after RNA binding and were thus able to use untagged RRM3 incubated with selected RNA ligands for structural studies of the complex. This allowed us to perform a comprehensive and comparative analysis of RRM3 interaction with three distinct RNA ligands: AUUUA motif-containing (AU6tnf), a U-rich motif with sparsely distributed adenosine residues (AU15) and a poly-U motif (U6). The complexes were crystallized and diffracted to 1.35, 1.90 and 2.01Å, respectively (see Table 1 for structural statistics, Supporting Information and Supplementary Figure S3A). In the case of AU15, electron density for seven nucleotides (UUUAUUU) could be fitted. The RNA nucleotides are recognized by a combination of base stacking, polar and hydrogen bond-mediated interactions. Three central bases in the RNA ligands (positions 2–4) mediate numerous contacts with RRM3, while the neighboring nucleotides show additional but fewer and less specific interactions (Figure 2A, Supplementary Figure S3B). Two consecutive uridines in position 2 and 3 contact RNP2 residues (Figure 2B). The U2 base (position 2) is stacked with Tyr249 and forms hydrogen bonds with Glu316. The U3 (position 3) is stacked with Phe247 and a network of hydrogen bonds is formed between the nucleotide and the C-terminus of RRM3 (Lys320, Thr321, Lys323). The recognition of the next nucleotide (position 4) is less specific. While in the structure of RRM3 with U6 and AU15, a uridine is recognized, in the case of AU6tnf RNA, an adenosine is bound in this pocket. This lower specificity is explained by fewer and less specific hydrogen bonds, and suggests that a G or C might also be tolerated in this position (see below). Nucleotides in positions 1, 5 and 6 form additional interactions (Figure 2C; Supplementary Figure S3B). However, the contacts observed depend on the respective RNA and are less involved than those seen for positions 2–4. In RRM3-AU6tnf and -AU15, the ribose in position 1 forms a (water-mediated) hydrogen bond with the main chain carbonyl of Lys285. On the 3′ end of the RNA, nucleotides in positions 5 and 6 are closer to the RRM3 β2β3 loop in case of the U6 ligand than when AU15 or AU6tnf are bound. In all three ligands, U6 is stacked with Phe279. To summarize, the core of the RNA interactions with RRM3 is formed by three binding pockets with canonical aromatic residues from RNP2 and RNP1 (Phe247, Tyr249, Phe289) that recognize a U–U–U/A RNA sequence motif. Additional, less important and less specific interactions are observed with flanking nucleotides.

### NMR analysis of the RRM3–RNA interaction

**Figure 3.**
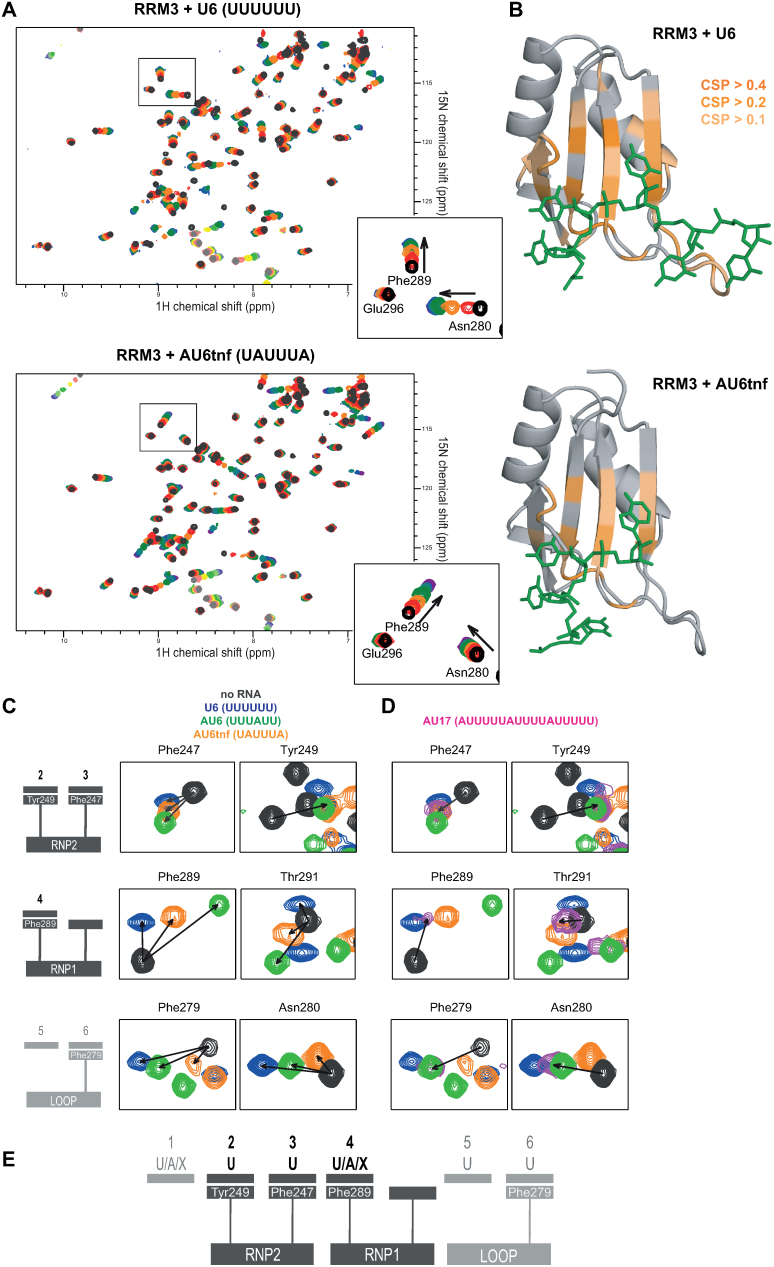
RNA binding by RRM3 in solution. (**A**) ^1^H,^15^N-HSQC spectra of free 120 μM HuR RRM3 (black) titrated with increasing amounts of RNAs–U6 and AU6tnf (5 titration points from 1:0.25 to 1:4.5 (U6) and 1:9.0 (AU6tnf)). Arrows in zoomed views indicate the direction of chemical shift perturbations (CSPs). (**B**) The CSPs induced by U6 and AU6tnf RNA binding are mapped on the structures of RRM3-U6 and RRM3-AU6tnf complexes. Colors indicate the extent of CSPs. (**C**) Zoomed views of ^1^H,^15^N-HSQC experiments for free and 6-mer RNA-bound RRM3. Arrows indicate the direction of CSPs. The selected residues are from three RNA-binding areas of RRM3 (RNP1, RNP2 and additionally the β2β3 loop - see diagrams on the left). (**D**) ^1^H,^15^N-HSQC spectra of RRM3 bound to AU17 were added to the zoomed views from (C). (**E**) Proposed consensus RNA recognition sequence.

We next performed NMR titrations using ^1^H,^15^N correlation experiments in order to validate the interactions seen in the crystal structure in solution and to probe the specificity of RNA binding in position 4. Increasing amounts of selected RNAs (U4, U6, AU6tnf, AU6, GU6 and GAGCAC as negative control) were added to ^15^N-labeled untagged RRM3 domain (Figure 3A, Supplementary Figure S4A). When U6 RNA was titrated, we observed binding kinetics that is fast (resonances shifting with increasing concentration of the ligand) and intermediate/slow (resonances of the free form disappearing and new resonances of the RNA-bound form appearing upon RNA addition) on the NMR chemical shift time scale (51) between the RNA-bound and RNA-free form. This is consistent with the micromolar affinities determined by ITC experiments (Table 2, Supplementary Figure S2A). In the case of AU6tnf, AU6 and GU6 the binding is predominantly in fast exchange, indicative of a weaker interaction. The binding of a U-rich RNA with a guanosine substituted into the position that is expected to be recognized in the RNP1 pocket of RRM3, confirms that this pocket can accommodate nucleotides other than U and A. The RNA ligand GAGCAC does not induce significant spectral changes (Supplementary Figure S4A). In contrast, while shorter (U4) and longer (U9 and AU17) RNAs show chemical shift perturbations (CSPs), thus confirming RRM3 specificity for U- and AU-rich RNA ligands (Supplementary information, Supplementary Figures S4B and S5). CSPs induced by U6 and AU6tnf are plotted onto the crystal structures of RRM3-U6/AU6tnf (Figure 3B). For both RNAs, affected areas included the β-sheet surface of the RNA and the C-terminus, consistent with the crystal structures (Figure 2). Interestingly, the β2β3 loop that is in close proximity to the 3′ end of the RNA in the crystal structure of the RRM3–U6 RNA complex appears to be most significantly affected by U6 titration (Figures 2C and 3B, Supplementary Figure S5). We therefore conclude that the recognition mode inferred from the crystal structures reflects the interactions in solution.

In order to further analyze the RNA binding preference of RRM3, we focused on NMR CSPs of selected residues from key binding regions (RNP1, RNP2 and the β2β3 loop) upon addition of three RNAs that present adenosine bases at specific positions of the RNA (UUUUUU, UUUAUU, UAUUUA) (Figure 3C, Supplementary Figures S4 and S5). RNP2 Tyr249 shows identical CSPs irrespective of the titrated RNA, while for RNP2 Phe247 small differences in the direction of the chemical shift changes are observed, probably reflecting its spatial proximity to RNP1 Phe289 (Figure 2A). The latter shows significant RNA-dependent differences in the CSP consistent with a less specific binding pocket. It is interesting to note that the CSPs in the loop area increase with increasing U:A ratio in the RNA ligand. We also incubated RRM3 with AU17, a longer RNA ligand from c-fos 3′UTR that contains single adenosine residues between longer stretches of Us and analyzed the NMR spectral changes (Figure 3D, Supplementary Figure S4B). We expected three possible outcomes: (i) if U is preferred in position 4, the CSPs would be identical to RRM3-U6; (ii) if A is preferred in position 4, the CSPs would be identical to RRM3-AU; (iii) if there is no preference for A or U, and/or the RNA shows a dynamic binding with multiple registers onto the RRM3 binding surface, the chemical shift perturbations should be inbetween U and AU-induced changes. The results provide evidence that there is no significant preference for A or U binding in position 4, as the chemical shifts of NMR signals in the RRM3-AU17 complex are always close but never overlapping with those of RRM3–U6 (Figure 3D, magenta). Both crystallographic and NMR data indicate that RNP2 residues specifically recognize uracils, while the RNP1 provides a binding pocket where other nucleotides (e.g. purines) can be accommodated (Figure 3E).

### Contribution of RRM3 to RNA binding of full-length HuR

In order to validate the structural data further, we used ITC to measure the affinities of wildtype (WT) and mutant RRM3 and full-length HuR titrated with various RNA ligands (U6 for RRM3 and AU17 for full-length HuR) (Table 2; Supplementary Figure S2D and E). We mutated the RNP1 and RNP2 motifs of RRM3 in both RRM3 and full-length HuR by replacing the aromatic residues with alanines and creating F287/F289A and F247/Y249A mutants, respectively. In case of RRM3, the F287/F289A mutant could not be purified due to insolubility. We additionally mutated Phe279 from the β2β3 loop to determine its potential role in RNA binding by RRM3. U6 RNA was used for RRM3 titrations as the high change of binding enthalpy allowed for the best detection of potential differences between wildtype and RNA binding mutants of RRM3. For HuR, we selected a U-rich RNA sequence derived from c-fos 3′-UTR that is expected to accommodate all three RRMs in a 1:1 complex (37,52). Notably, the F247/Y249A mutation in RRM3 completely abolished its RNA binding capacity, while in the case of full-length HuR, the *K*_d_ increased by more than 8-fold (from 40 nM to 351 nM). The RNP1 mutation (F287/F289A) in full-length HuR increased the *K*_d_ by >4-fold (194 nM), while the F279A mutation had only a moderate effect on RNA binding by RRM3 (*K*_d_ from 19 to 45 μM) and no effect on full-length HuR–RNA interaction (29 nM). Thus, the ITC data confirm a primary role of RNP2 and, to a lesser extent, of RNP1 for RNA binding by RRM3, as well as a marginal contribution of the Phe279 in the β2β3 loop. Moreover, our data clearly demonstrate that RRM3 contributes to RNA binding by full-length HuR *in vitro*.

### RRM3 mediates HuR dimerization

**Figure 4.**
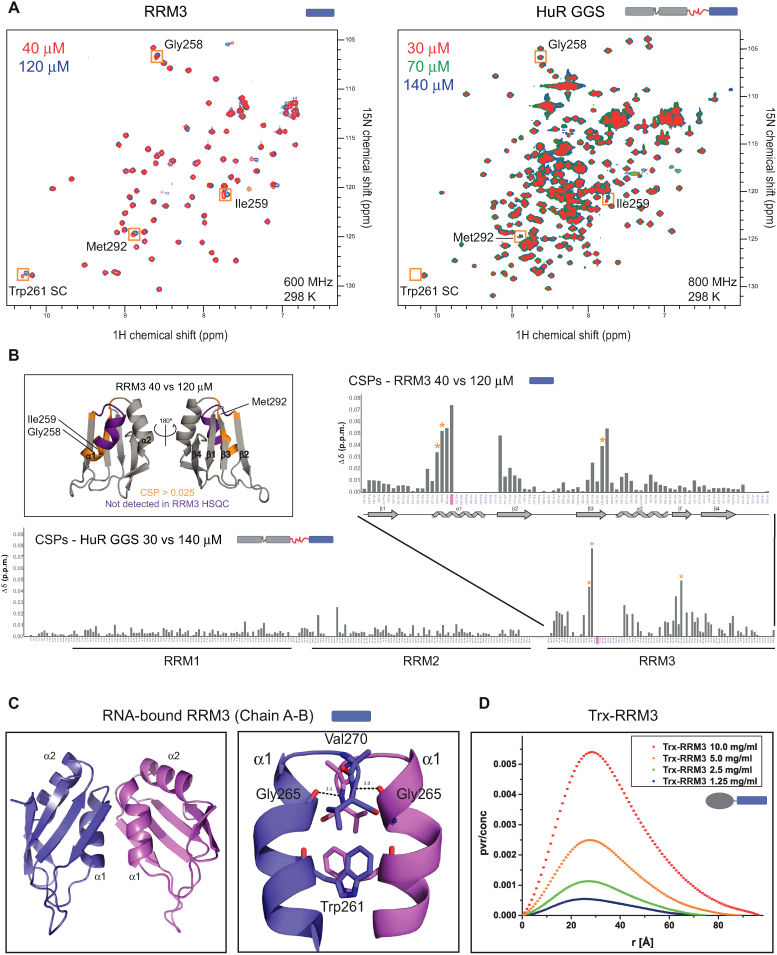
Concentration-dependent dimerization of HuR via RRM3. (**A**) Superposition of ^1^H,^15^N-HSQC spectra of HuR RRM3 and full-length HuR GGS at different concentrations as indicated. Examples of concentration-dependent CSPs for both RRM3 and HuR-GGS are highlighted by orange boxes with the corresponding assignment indicated. The Trp261 side chain is not detected in HuR GGS HSQC. (**B**) CSP values calculated based on comparing lowest and highest concentration of RRM3 and HuR GGS are plotted versus residue number. Residues that are not observable in NMR spectra of RRM3 at any tested concentration are marked in purple. The asterisks indicate the strongly affected residues that are highlighted by orange boxes in the NMR spectra in (A). Trp261 is highlighted in magenta. The localization of secondary structures in RRM3 is indicated below the graph for RRM3. **Boxed area**. Cartoon representation of RRM3 structure with absent residues marked in purple and concentration-affected residues (CSP > 0.025) marked in gold. (**C**) Cartoon representations of adjacent RRM3 chains in the crystal structures of RNA-bound RRM3 and zoomed view of the dimer interface with hydrogen bonds marked as dashed lines. (**D**) SAXS P(*r*) curves of increasing concentrations of Trx-RRM3 (1.25–10 mg/ml).

The dimerization of HuR RRM3 was proposed to be mediated by Trp261, a surface exposed residue conserved in the RRM3 of HuR paralogues and orthologues, but absent in other RRMs that are structurally homologous to HuR RRM3 (Figure 1E) (40). Nevertheless, structural details of the dimerization interface are not known. The analysis of free Trx-RRM3 and RNA-bound RRM3 crystal structures suggests several possible dimerization interfaces, three of them involving Trp261 (Supplementary Figure S6A–D). In order to determine the dimerization interface in solution, in the absence of crystal packing effects, we acquired NMR spectra at different concentrations of RRM3 alone and of a variant of full-length HuR where the linker connecting RRM2 and RRM3 is replaced with Gly-Gly-Ser repeats (HuR GGS) (Figure 4A). HuR GGS was used instead of HuR WT, because it permitted the analysis of a concentration series (30–140 μM) that was not feasible with HuR WT. For both RRM3 and HuR GGS proteins, residues 262–270 from helix α1 and loop α1β2 are not observable in the NMR spectra, irrespective of concentration, while a number of residues show concentration-dependent shifts. This is indicative of a dynamic equilibrium between dimeric and monomeric forms where the affected residues are located at the dimerization interface. Concentration-dependent chemical shift perturbations were therefore calculated for both RRM3 and HuR GGS and plotted against residue number (Figure 4B). Large CSPs cluster in two areas: (i) around the residues 262–270, i.e. at the beginning of helix α1 and strand β2 and (ii) in the loop following the β3 strand. The affected area correlates best with the dimeric interface found between chains A and B in the structures of RNA-bound RRM3 (Figure 4C) suggesting this is the dimerization interface in solution. Notably, our NMR titrations of HuR GGS show that concentration-dependent CSPs only affect RRM3, indicating that there is no additional dimerization interface present in this almost full-length HuR protein (Figure 4A and B).

We used SAXS experiments to characterize the dimeric state of RRM3 and full-length HuR in solution. RRM3 alone was not suitable for SAXS analysis due to radiation damage and the Trx-RRM3 fusion protein was used instead. Trx-RRM3 showed significant concentration-dependent scattering in solution (Figure 4D), consistent with a dimerization of RRM3 with micromolar affinity. Note, that *E. coli* Trx 1 is monomeric in solution and is thus not expected to contribute to dimerization of the Trx-RRM3 fusion protein (53). SAXS data recorded for Trx-RRM3 at the lowest tested concentration could be fitted to the crystallographic structure of Trx-RRM3 dimer (Supplementary Figure S1A) with χ^2^ = 1.17 (Supplementary Figure S6E). We then analyzed the dimerization of the untagged full-length HuR comparing the WT and W261E proteins (Supplementary Figure S6F). SAXS data of HuR WT are concentration-dependent, with an apparent molecular weight that increases with protein concentration. The apparent molecular weight is much higher than expected for a monomer (37 kDa) even at the lowest tested concentration (0.5 mg/ml–105 kDa), indicating a formation of dimeric and higher order oligomeric states with increasing protein concentration. In contrast to HuR WT, SAXS data of HuR W261E at varying concentrations are very similar, consistent with the expectation that the W261E mutation in RRM3 abolishes HuR dimerization. The estimated molecular weight (40–45 kDa) is consistent with the monomeric protein. To conclude, HuR full-length exists in a dynamic equilibrium between monomeric and dimeric/oligomeric states in solution. This equilibrium is strongly shifted towards the oligomeric forms with increasing protein concentration. Trp261 on the α-helical side of RRM3 is a key mediator of the dimerization and its mutation significantly decreases the dimerization and the oligomerization, not only of RRM3 (40), but also of HuR full-length *in vitro*.

### Structural features of full-length HuR and HuR-RNA interactions

**Figure 5.**
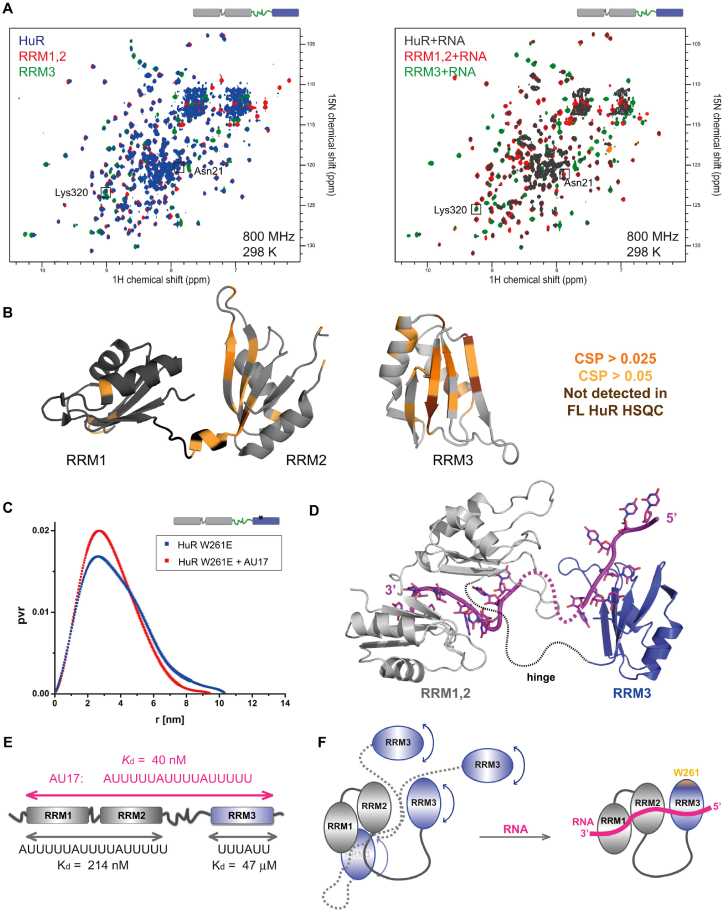
Domain arrangements in free and RNA bound HuR. (**A**) Overlays of ^1^H,^15^N TROSY spectra of free and RNA-bound deuterated HuR with ^1^H,^15^N HSQC spectra of free and RNA-bound deuterated RRM1,2 and RRM3 (protein:RNA ratio = 1:1.1). The following RNAs were used: AU17 for HuR, AU12 for RRM1,2 and U6 for RRM3. Examples of amide NMR signals of residues involved in RNA binding are enclosed in squares. (**B**) CSPs were calculated comparing spectra of free HuR and individual domains (RRM1,2 and RRM3) and are mapped on the structures of RRM1,2 (PDB ID: 4EGL) and RRM3 (PDB ID: 6G2K). Signals that were line-broadened (‘not detected’) in the spectra of full-length HuR are also indicated. Peaks of full-length HuR that could not be unambiguously assigned were not considered in the analysis. (**C**) Overlay of SAXS-derived P(*r*) curves of free and RNA-bound HuR W261E. (**D**) Structural model of AU17-bound HuR based on rigid body modelling of the RRM1,2-RNA (PDB 4EGL) and RRM3-RNA (PDB 6G2K) crystal structures based on SAXS data of the HuR-RNA complex. See Supplementary Figure S10 for details. (**E**) All three RRM domains contribute to RNA binding, as indicated by the 5-fold increase of RNA binding affinity of full-length HuR compared to RRM1,2. (**F**) Schematic picture indicating the suggested mechanism of RNA binding by HuR. Free HuR exhibits a dynamic arrangement of RRM3 and the RRM1,2 tandem domains with transient interactions of RRM3 with RRM1,2 as indicated by NMR and SAXS data. Upon recognition of ARE RNA all three domains contact the RNA forming a rigid assembly.

We used NMR and SAXS experiments to characterize the multidomain arrangements of the three RRMs in HuR free and bound to RNA. The analysis of full-length WT HuR was hampered by its low solubility (<1.5 mg/ml) and oligomerization. Therefore, in addition to WT HuR, we also used its two mutants: HuR W261E (as monomeric protein) and HuR GGS (to increase solubility and stability) (Figure 1B). We confirmed that the affinities of the two HuR variants to AU17 RNA are virtually identical to HuR WT using ITC (Table 2, Supplementary Figure S2F and G), thus validating that these HuR variants can be used to study the domain arrangement of RNA-bound HuR. First, we monitored changes in free full-length HuR spectra as compared with spectra of RRM1,2 and RRM3 (Figure 5A). While many signals superimpose well, some spectral changes (chemical shift perturbations and line-broadening) are observed for residues located on the β-sheet surfaces of RRM2 and RRM3 and in the linker connecting RRM1,2 (Figure 5A and B). This suggests that RRM3 and/or the RRM2–RRM3 linker may transiently interact with RRM2. Given that similar (although weaker) spectral changes are also observed in the context of the HuR GGS protein that lacks the hinge region these data suggest that RRM3 may be at least transiently in spatial proximity to RRM2 (Supplementary Figures S7A, B and S8A–C). This is further confirmed by paramagnetic relaxation enhancement (PRE) data obtained with HuR RRM1-3 (aa 18-323) spin-labeled at the single surface-exposed Cys245 residue localized on the β-sheet surface of the RRM3 (Supplementary Figures S7C, S8D-F). The spin label induces line-broadening and CSPs in the same region comprising the β-sheet surface of RRM2, confirming a direct effect. Additional, but fewer changes map to the helical face of RRM1. This suggests that the β-sheet side of the RRM2 (and to a lesser extent the helical side of RRM1) can be in close spatial proximity to the Cys245-containing β-sheet side of the RRM3. Thus, although RRM1,2 and RRM3 tumble mostly independently in the absence of RNA, weak and transient contacts between these two modules are present in solution, which may support cooperative RNA binding by all three domains.

To further assess the domain arrangements of all three RRM domains, we analyzed NMR data of full-length HuR free and when bound to RNA (Figure 5A). The latter spectrum shows significant line broadening and complexity impeding unambiguous assignment of many NMR signals. While complete assignment of this spectrum is challenging, we do observe that NMR signals of residues from the spectrum of RNA-bound full-length HuR superimpose well with those of both RRM1,2 and RRM3 bound to RNA (Figure 5A, right). This indicates that all three RRMs interact with the RNA and that details of the interactions observed in the RRM1,2 and RRM3 RNA complexes are conserved in the full-length HuR. For further analysis, we compared NMR spectra of the ternary complexes with differentially labelled separate RRM1,2 and RRM3 proteins together with RNA. For this, chemical shifts were monitored for ^15^N-labeled RRM3 bound to AU17 RNA in the presence and absence of unlabeled RRM12, and of ^15^N -labeled RRM1,2 bound to AU17 RNA in the presence and absence of unlabeled RRM3. In these experiments, each spectrum shows only residues from the ^15^N-labeled part of HuR (RRM1,2 or RRM3) and the signals undergoing spectral changes can be unambiguously identified (Supplementary Figure S9). The affected residues map to the β-sheet faces of RRM2 and RRM3 and to the β2β3 loop in RRM3. Thus, a transient proximity of RRM2 and RRM3 that is preexisting in the absence of RNA (see previous section) may reduce the entropy loss associated with the formation of a rigid protein-RNA complex that involves binding of all three RRM domains to RNA.

To assess the relative tumbling of HuR RRM domains free and when bound to RNA, we compared ^15^N NMR linewidths based on measurement of ^15^N *T*_1ρ_ relaxation times. The average linewidths of each of the HuR RRMs are consistently decreased (by 1.6-, 2.1- and 1.6-fold for RRM1, RRM2 and RRM3, respectively) upon RNA binding indicating a formation of a more compact entity engaging all three RRMs in RNA binding (Supplementary Figure S10G). In conclusion, several NMR experiments indicate that the HuR RRM1,2 and RRM3 regions tumble rather independently in the absence of RNA, but that weak and dynamic interactions of RRM2 and RRM3 are enhanced upon binding to a target RNA, where all three domains contact the RNA ligand.

Finally, we applied size-exclusion chromatography in line with SAXS (SEC-SAXS), in order to obtain low-resolution structural information in solution for the monomeric full-length HuR (W261E). Data analysis was conducted using the ensemble optimization method (EOM) that is compatible with the existence of variable conformations of the protein. This is advised as the HuR RRMs are connected by unstructured flexible linker regions, consistent with our NMR data, and dynamic RRM arrangements in RRM1,2 that has been reported previously (38). The distribution of radii of gyration (*R*_g_) of the selected ensemble for HuR W261E shows a high frequency at small *R*_g_ compared to the random pool, indicating a prevalence of more compact structures (Supplementary Figure S10A). The ensemble distribution of the free HuR domains can be explained by four representative conformations (Supplementary Figure S10F), most sampling relatively compact arrangements, with few extended structures being present. Upon RNA binding, HuR W261E adopts a more compact and rigid conformation, as indicated by the *R*_g_, pairwise distance distribution P(*r*) and Kratky plots derived from the SAXS data (Figure 5C, Supporting information, Supplementary Figure S10A, C–E). As these data indicate a more homogenous globular domain arrangement, a structural model of HuR in complex with RNA was created using Coral (45) based on the crystallographic structures of RRM1,2 and RRM3 in complex with their respective RNA ligands (4ED5 and 6G2K). Based on the NMR data (Figure 5B, Supplementary Figures S8 and S9) and on the quality of the fit to the experimental SAXS curves, RRM3 binds upstream of RRM1,2 at the 5′ end of the RNA and in close proximity to RRM2 (Figure 5D, Supplementary Figure S10H). In conclusion, we show that the RRM domains of free HuR in solution exhibit a dynamic behavior with a significant population of compact conformations, the latter becoming stabilized upon RNA binding (Figure 5E and F).

### Role of HuR RNA binding for cellular function

**Figure 6.**
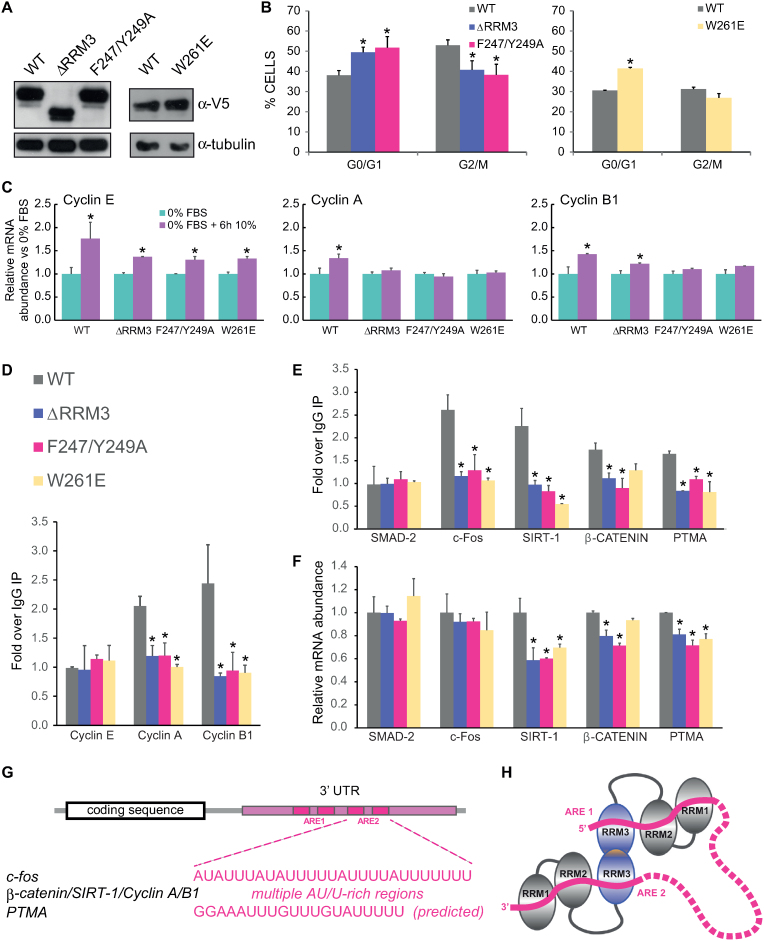
Contribution of RRM3 to HuR function in human RKO cells. (**A**) Protein levels of V5-tagged HuR constructs in RKO cells stably expressing HuR-V5 WT, HuR-V5 ΔRRM3, HuR-V5 F247/Y249A and HuR-V5 W261E, α-tubulin is shown as loading control. (B–F) Cells were serum starved for 16 h and subsequently cultured in serum for 6 h before being collected for experiments. (**B**) Flow cytometry histograms of cell cycle in RKO cells stably expressing HuR-V5 WT, HuR-V5 ΔRRM3, HuR-V5 F247/Y249A and HuR-V5 W261E. (**C**) Relative cyclin mRNA abundance in RKO cells after serum starvation and 6h after serum re-addition. Statistical significance was calculated between mRNA levels in stimulated versus starved RKO cells.**(D, E)** V5 (HuR) RNP IP. Immunoprecipitations were conducted with anti-V5 antibodies and with IgG to account for unspecific binding. The final results are normalized to IgG IP. (**F**) Relative mRNA expression levels of HuR targets and of SMAD-2 (negative control). (B–F) The experiments were repeated 3 times. The p values were calculated using two-tailed Student's *t*-test. * indicates statistically significant fold changes (*P* < 0.05). Error bars indicate standard deviations. (**G**) HuR targets exhibit multiple AU-rich elements (AREs) in their 3′ UTRs. (**H**) Model of RNA recognition of tandem ARE motifs by dimeric HuR.

We have shown that HuR RRM3 has significant RNA-binding activity *in vitro* and contributes to the RNA-binding affinity of the full-length HuR protein. To confirm this in a cellular context, stable RKO cell lines expressing comparable amounts of V5-tagged HuR WT, ΔRRM3, F2447/Y249A and W261E were created (Figure 6A). While the ΔRRM3 mutant lacks the RRM3 domain and may accumulate defects due to diverse proposed functions of RRM3 (RNA-binding, dimerization, interactions with binding partners), the F2447/Y249A mutation specifically targets the ability of RRM3 to bind RNA and W261E impedes the dimerization of HuR via RRM3. V5 is a small tag that allows for easy detection and immunoprecipitation of exogenous HuR constructs, but does not interfere with HuR function (6). Cell cycle analysis in the three cell lines showed lower proliferation of cells expressing HuR ΔRRM3, F2447/Y249A and W261E as compared with HuR WT (Figure 6B). As HuR is known to directly regulate cyclin A and cyclin B1 mRNA levels (54) we tested whether the levels of these mRNA increase after serum stimulation in RKO cells transfected with HuR WT or HuR with mutations in RRM3. Cyclin E does not exhibit an ARE in its 3′ UTR and is not bound by HuR, thus serving as a negative control (54). For cyclin E, we observed an increase in mRNA level irrespective of the overexpressed HuR variant. In contrast, cyclin A and B1 mRNA levels failed to increase when HuR RRM3 mutants were expressed (Figure 6C). In accordance with these findings, we observed no binding of HuR RRM3 mutants to cyclin A and B1 mRNAs in RNP-IP experiments. As expected, Cyclin E was not immunoprecipitated by either of the HuR variants (Figure 6D). HuR is also known to stabilize other mRNAs related to cell proliferation in RKO cells (55). We quantified both the levels of these mRNAs bound by exogenous HuR WT and RRM3 mutants and their total levels in cells (Figure 6E–F). We selected c-fos, Sirtuin 1 (SIRT-1), β-catenin and prothymosin alpha (PTMA), as HuR has been previously shown to positively regulate the stability of these mRNAs (52,55–57). SMAD-2 was used as a negative control, as HuR binding to SMAD-2 mRNA has not been reported. The RNP-IP (Figure 6E) clearly shows that for c-fos, SIRT-1, β-catenin and PTMA significantly less mRNA is bound by HuR ΔRRM3 and F2447/Y249A compared to HuR WT. Cells overexpressing V5-tagged HuR W261E also show reduced binding to mRNAs of c-fos, SIRT-1 and PTMA, while this is not observed with statistical significance for β-catenin mRNA.

The impaired binding of all HuR RRM3 mutants is reflected in reduced total mRNA levels in case of SIRT-1 and PTMA (Figure 6F). In contrast, total levels of β-catenin mRNA are only affected by the deletion of RRM3 or the F2447/Y249A mutation, which impairs RNA binding by RRM3, but not dimerization. The observed differences are small, but significant and consistent with the fact that endogenous HuR was not knocked-down in the stable cell lines and thus can exert its regulatory effect on the tested mRNAs. Surprisingly, no change in c-fos mRNA abundance was observed in the presence of different HuR constructs, even though the binding of its mRNA by RRM3 mutants is strongly decreased. This can be due to the action of the endogenous HuR and/or to other mechanisms controlling the levels of c-fos expression (58). As expected, SMAD-2 mRNA is not bound by HuR and its levels are not affected by HuR WT or mutants. Altogether, these data demonstrate that HuR RRM3 contributes to the binding of endogenous mRNA targets in human cells and that the decreased binding can lead to lower mRNA levels of HuR targets. In addition, our findings show that RRM3 dimerization enhances HuR binding to ARE-containing mRNAs.

## DISCUSSION

The C-terminal RRM of HuR and other ELAVL proteins is their most conserved domain. Yet, it has been poorly understood how RRM3 contributes to HuR function. Although RNA binding and enzymatic activity have been previously reported, the main role of RRM3 is thought to be contributing in the assembly of HuR oligomers on target mRNAs and its potential regulation by posttranslational modifications (5,6,10,37,40,59). Here, we present the crystal structure of HuR RRM3 in its apo and RNA-bound forms. We reveal molecular details of both the dimerization interface and RNA recognition. The latter, together with further biochemical and functional analyses, provide clear evidence that RRM3 contributes to HuR RNA binding both *in vitro* and *in vivo*. Our NMR and SAXS data indicate that RNA binding by all three HuR RRMs induces a rigid and more compact multidomain arrangement that is required for functional activity *in vivo*. Given the 5-fold increased binding affinity of full-length HuR compared to RRM1,2 (Figure 5E) and the importance of RRM3 dimerization for function (Figure 6), we propose that dimerization enables recognition of tandem AREs involving all RRMs by dimeric HuR (Figure 6G and H).

### RNA specificity of RRM3 binding

The recognition of RNA by RRM3 is consistent with general characteristics of RNA binding by RRM domains. The single stranded RNA is bound on the β-sheet surface of the domain, with principal interaction areas involving RNP residues. The described involvement of C-terminal residues in RNA recognition is also common for RRM domains (Figure 2) (60,61). In RRM3, the major and most specific interaction areas involve aromatic residues from the RNP2 motif, and their substitution (F247A/Y249A) completely abolishes RNA binding by RRM3 (Table 2 and Supplementary Figure S2). Such involvement of both aromatic residues from the RNP2 is not frequent in RRMs, but it is also described for CUG-binding protein 1 (CUG-BP1/CELF1) RRM3 (47). The single RNP1 pocket comprising Phe289 has the weakest contribution of hydrogen bonding and is therefore least specific.

Crystal structures, ITC and NMR titrations show a preference of RRM3 for U-rich (UUUUUU and UUUAUU) over AU-rich (UAUUUA) targets. This binding preference matches very well with the major binding motifs, (i) identified by PAR-CLIP for full-length HuR *in vivo* (UUUUUUU, UUUAUUU, UUUGUUU, stretches of either poly-U or of three to four Us separated by an A or C nucleotide) (14,15,62), (ii) discovered by RNAcompete *in vitro* (- UUUUUUU, UUUGUUU, UUAUUUU, UUUAUUU, UUGUUUU) (63), (iii) and computational analysis of HuR targets based on microarray analysis of HuR immunoprecipitates (13). The increased binding affinity observed with increasing length of the poly-U ligands in our *in vitro* studies, suggests that binding in multiple binding registers could greatly enhance the apparent binding affinity with extended poly-uridine ligands when compared to AU-rich targets. The preference for U-rich targets is consistent with similarities in RNA recognition by HuR RRM3 and RRM1. The sequence and the direction of the single-stranded RNA ligand bound by RRM3 and RRM1 (PDB ID: 4ED5) in positions 1 to 4 are comparable (U/A–U–U–U/A versus A–U–U–U) (Supplementary Figure S11A). Stacking interaction and hydrogen bonds with structurally equivalent amino acids are formed in position 1 and 2. The third binding pocket is formed by RNP2 in RRM3 and RNP1 in RRM1. There is a comparable extensive hydrogen bond network with residues from the C-terminus of RRM3 and the linker sequence following RRM1. The fourth binding area is marked by structurally equivalent phenylalanines from RNP1 (Phe65 and Phe289). The hydrogen network being distinct, the specificity of this pocket differs for RRM1 (U/C) and for RRM3 (U/G/A and probably C). In contrast to RRM3, the RRM1–RNA interaction is strengthened by additional hydrogen bonds between the RNA and RRM2. Thus, the structural similarities between RRM1 and RRM3 lead to similar RNA-binding specificity. However, the affinity of RRM1 is higher due to the presence of additional hydrogen bonds and contributions from RRM2 in the context of the RRM1–RRM2 tandem domains, which bind cooperatively to a larger RNA motif (28).

### HuR dimerization involves only RRM3

Hu/ELAV proteins have been reported to dimerize and multimerize on RNA targets with the participation of RRM3 and of the hinge region (33,37,64). The molecular basis of the contribution of the hinge region is unknown. In case of *D. melanogaster* ELAV RRM3, three short sequences responsible for RRM3 dimerization *in vitro* were identified (33). In HuR RRM3, they correspond to Asp254 - Glu257, Trp261 and Met292–Tyr295. Tyr261 was later confirmed to disrupt HuR RRM3 dimerization (40). Here, we further show that the W261E mutation strongly reduces the dimerization and oligomerization of full-length HuR *in vitro* (Supplementary Figure S6F). Moreover, our crystal structures of RNA-bound RRM3, together with NMR experiments indicate that the dimerization interface is formed by stacking of Trp261 and hydrogen bonds between amino acids from helix α1 and the α1–β2 loop (Figure 4). While Diaz-Quintana *et al.* (65) proposed a model in which one RRM3 domain bound to the RNA-binding β-sheet of the other subunit in an almost perpendicular orientation, our data fit better with the previously reported antiparallel orientation of the RRM3 monomers (40). We also do not rule out the existence of additional contributions to dimerization, e.g. involving residues of the RRM2–RRM3 linker. In any case, the RRM3 dimerization interface involving Trp261 has the strongest contribution and mediates an initial dimerization event that can potentially be further stabilized by additional contacts. This is consistent with the molecular weight of HuR estimated from SAXS measurement, which approaches the value expected for a dimer at low concentrations but corresponds to oligomeric species at higher concentrations, while HuR W261E shows a molecular weight corresponding to a monomeric protein at all concentrations (Supplementary Figure S6F). The presence of oligomeric species might play a role for spatial organization of HuR on long RNA targets and contribute not only to enhance RNA-HuR affinities, but also to structurally orchestrate the recognition of ARE-RNA targets. HuR dimerization through RRM3 could provide an anchor point to bring close AREs that are distant from each other in sequence. A similar rationale was described in the KSRP protein where the rearrangement of its central KH domains was related to the context of the highly structured 3′UTRs (66).

### Multidomain arrangement of free and RNA-bound HuR

Multidomain proteins comprising flexible linker regions are often dynamic and endowed with conformational freedom (67–70). Our NMR and SAXS data of full-length HuR indeed suggest that the three RRMs of HuR tumble independently and that the free protein adopts multiple conformations, ranging from compact to extended. The analysis of NMR chemical shift differences when comparing spectra of full-length HuR and single domains suggests that there are weak and transient contacts between RRM2 and RRM3. When RNA is bound, both SAXS and NMR data indicate that all three domains form a more compact entity as they exhibit an increased and comparable molecular tumbling correlation time and a more compact pairwise distance distribution as seen by the SAXS P(*r*) data (Supplementary Figure S10G; Figure 5C). It is plausible that the dominant HuR RNA binding domains (RRM1,2) make the initial contact with the RNA. Subsequently, RRM3, connected by the 60-amino acid flexible hinge region, could bind the RNA both 5′ or 3′ to the RRM1,2 binding site.

### Mutational analysis highlights the role of RRM3 in HuR cellular function

Albeit RNA recognition by RRM3 is of moderate strength (19 to 156 μM depending on RNA target), it contributes to RNA binding of full-length HuR, as the mutation in RRM3 RNP2 (F247/Y249A) significantly increased the *K*_d_ of HuR interaction with c-fos 3′UTR RNA (AUUUUUAUUUUAUUUUU) (Supplementary Figure S2 and Table 2). This argues that the extent of the contribution of RRM3 to RNA binding depends on the specific type of ARE. Based on our structural and biophysical data we predict that the contribution is greater for ARE motifs of class I or III (AUUUA motifs dispersed within U-rich regions or U-rich regions without AUUUA motifs). For these AREs, avidity effects with poly-U regions can increase the binding contributions by RRM3 to the overall affinity and thus enhance HuR functional activity. In contrast, contributions of RRM3 to binding and functional activity of HuR are expected to be lower for class II AREs (exhibiting different patterns of repeated and overlapping AUUUA motif, but lacking poly-U stretches). This could explain why RRM3 has only a modest effect on HuR binding to class II ARE of TNF-alpha (e.g. AUUAUUUAUUUAUUUA) *in vitro* (37).

Importantly, we detected significantly impaired binding of HuR Y247/F249A to several well-established mRNA targets in human RKO cells (Figure 6D–E). This reduced binding was reflected in reduced levels of those mRNAs in cells expressing HuR Y247/F249A, presumably due to their defective stabilization caused by less efficient HuR binding (Figure 6F). The concurrent presence and action of endogenous HuR in those cells may explain why the observed effect is weak. Importantly, to our knowledge, this is the first direct assessment highlighting the role and contributions of RRM3 to RNA binding and functional activity by HuR in human cells, as previous experiments so far have used deletions of the entire RRM3 (34,35).

Interestingly, the disruption of the RRM3 dimerization interface (HuR W261E) also resulted in decreased binding of full-length HuR to some cellular targets. Our *in vitro* data indicate that the binding affinity of HuR W261E to an RNA target with a single HuR binding site is not decreased. Nonetheless, targets that exhibit two or more HuR binding sites can be more efficiently recognized by dimeric/oligomeric HuR complexes (Figure 6H), thus explaining the reduced binding and activity of the W261E mutant *in vivo*. Additionally, dimerization and potential oligomerization may increase the local concentration of HuR and thus enhance its functional activity. The spatial separation of the RNA binding site and the dimerization interface in RRM3 structure allows this domain to be simultaneously involved in both RNA- and protein-protein interactions.

Recently, it has been reported that RRM3 may exhibit posttranslational modifications. For example, Lys283, Lys313 and Lys326 are neddylation sites, while Ser304 and Ser318 are phosphorylation sites (6,10,36). These modications were shown to affect stability, intracellular localization and RNA binding of HuR. Our crystal structure of RRM3 reveals that the neddylation sites (Lys283, Lys313) are localized and directed away from the main RNA binding and dimerization site (Supplementary Figure S11B). We speculate that the covalent attachment of NEDD8 could stabilize RRM3 or alter HuR interaction with other proteins, without disturbing its RNA-binding and dimerization capacity. The phosphorylated Ser318 is in spatial proximity to the RNP2 sequence motif and it is conceivable that the negative charge and steric bulk of the phosphate group could modulate the association of HuR with RNA targets. The other modified Serine (Ser304) is remote from the RNA-binding and main dimerization interface and its phosphorylation could thus affect HuR function by altering its association with protein binding partners. Investigating these RRM3 modifications in the context of full-length HuR *in vitro* and *in vivo* will be an interesting topic for future investigation.

## CONCLUSIONS

We have studied the overall structure and domain arrangements of HuR with a particular focus on the role of RRM3 and its contribution to full-length HuR function. (i) Using high-resolution structural analysis and *in vitro* binding studies we demonstrate that RRM3 is a *bona fide* RNA binding domain with a canonical mode of RNA recognition expected for RRM domains. Residues centered around RNP2 and RNP1 motifs specifically recognize a UUU/A RNA sequence. (ii) Mutation of key residues involved in RNA recognition reduces the RNA binding capacity of full-length HuR both *in vitro* and in human cells. (iii) We identified structural details of the HuR dimerization interface localized on the α-helical surface of RRM3 and thus spatially separated from the RNA-binding region. The interaction is mediated by the conserved Trp261 residue. (iv) Our analysis of the full-length HuR conformation in solution indicates that the three RRMs are flexibly arranged in the absence of RNA, while RNA binding induces a more rigid domain arrangement where all three RRMs contribute to RNA binding. (v) Finally, we demonstrate that RNA binding by RRM1,2 together with RRM3 and the dimerization involving Trp261 in RRM3 are required for functional activity of HuR *in vivo*.

## DATA AVAILABILITY

Atomic coordinates and structure factors for the reported crystal structures have been deposited with the Protein Data bank under accession numbers 6GD1 (RRM3-Trx), 6GD2 (RRM3-AU15), 6GD3 (RRM3-AU6tnf) and 6G2K (RRM3-U6). Amide NMR chemcial shifts have been deposited with the BMRB with accession codes 27669 (HuR RRM1-3), 27670 (HuR GGS), and 27671 (RRM3 alone).

## Supplementary Material

Supplementary Data
