# Supplementary material for: HuR biological function involves RRM3-mediated dimerization and RNA binding by all three RRMs
Source: Nucleic Acids Res. 2018 Nov 10;47(2):1011–29. doi: 10.1093/nar/gky1138 (PMC6344896; doi:10.1093/nar/gky1138)
Supplement: Supplementary Data [file gky1138_supplemental_files.pdf]

SUPPLEMENTARY DATA

HuR biological function involves RRM3-mediated dimerization and RNA binding by all three RRMs

Marta Pabis[1,2,3], Grzegorz Popowicz[1,2], Ralf Stehle[1,2], David Fernández-Ramos[4,5], Sam Asami[1,2], Lisa Warner[1,2], Sofía M. García-Mauriño[6], Andreas Schlundt[1,2], María L. Martínez-Chantar[4,5], Irene Díaz-Moreno[6], Michael Sattler[1,2,*]

Supplementary Results

Crystal structure of Trx-RRM3

The structure of Trx-RRM3 fusion protein was determined by molecular replacement using the structure of *E. Coli* thioredoxin 1 (2TRX) as a search model. The asymmetric unit contains two copies of the fusion protein, where two RRM3s are sandwiched between two Trx domains **(Supplementary Figure 1A).** Several hydrogen bonds between RRM3 and Trx presumably help to enhance the stability and solubility of RRM3 **(Supplementary Figure 1B)**. The refined RRM3 model contains residues Gly243 to Thr321. The C-terminal amino acids (322-326) were not modeled owing to lack of electron density.

RNA binding of RRM3 - calculation of minimal binding site

In order to determine if the higher affinity of RRM3 for the U6 ligand represents an apparent increase in affinity due to multiple binding registers, we measured the affinity constants $K_a^{(app)} = 1/K_d$ of U6, U5 and U4 RNAs **(see table below)** and applied the equation from (Kelly, 1976):

$$K_a^{(app)} = (l-m+1)*K_{intr}$$

where $K_a^{(app)}$ is the observed equilibrium binding constant, K_{intr} is the intrinsic affinity of the minimal binding site, *l* is the ligand length and *m* is the minimal ligand length.

For two ligands of different length and K_d (U5: l = 5; K_d = 34 and U6: l = 6; K_d = 16) (Table 3):

$$K_a^{(app)} (l=5)/K_a^{(app)} (l=6) = (6-m)*K_{intr} /(7-m)*K_{intr} = (6-m)/(7-m).$$

Since $K_d = 1/K_a$, then binding modes, m, of two, three, four and five nucleotides will have apparent K_d(U6)/K_d(U5) ratios of 4/5 (=0.80), 3/4 (=0.75), 2/3 (=0.67) and 1/2 (=0.50), respectively (**Supplementary Figure 2B**). The ratio of experimentally observed K_d values for the U5 and U6 oligonucleotides (0.47; see Table below) suggests a minimum binding site of five nucleotides (*i.e.* closest to 1/2). Thus, the higher affinity of RRM3 for U6 as compared with AU6tnf may reflect multiple binding registers. A binding site harboring five nucleotides is in agreement with the very inefficient binding of the U4 RNA (where the low heat release precluded K_d determination) (**Supplementary Figure 2B**).

Protein	RNA	sequence	K_d	n
RRM3	U6	UUUUUU	15.8 µM ± 1.5	0.6
RRM3	U5	UUUUU	33.9 µM ±12.2	0.6
RRM3	U4	UUUU	Not determined	

Crystal structures of RNA-bound RRM3

The three crystals of RRM3 bound to RNA share a similar organization of the asymmetric unit with three RRM3 chains: two RRM3 molecules interacting with RNA and a single unbound RRM3. The RNA in the asymmetric unit was fitted as a single chain bound by two RRM3 molecules. The electron density spans six nucleotides in case of U6 and AU6tnf and seven in case of AU15 **(Supplementary Figure 3A)**. In case of RRM3-AU6tnf complex, the electron density of the RNA presented several unclear areas, even though the resolution of the crystal was very high (1.35 Å). This probably results from (i) weaker binding due to suboptimal sequence of the RNA and (ii) more than one possible binding register. The following difficulties were encountered. (i) Next to chain 1 only two nucleotides are fitted (position 3 and 4 - see diagram in Figure 2). There is a weak electron density where an additional nucleotide could be fitted in position 2, but the same electron density fits better to the side chain of Phe279 from an adjacent RRM3 molecule and this option was chosen in the final model. (ii) The third nucleotide from the 5' end that is not involved in stacking interactions (position 1) has uneven electron density (good for the phosphate bond but only partial for the rest of the nucleotide) This is possibly resulting from its mobility or because in a subset of asymmetric units the RNA is not continuous, but each RRM3 binds a distinct RNA molecule. The lower electron density of this nucleotide impedes unambiguous assignment as A or U. (iii) The fit of A in position 4 is not as good and clear as of the U in position 2 and 3. Nevertheless, the fit is better when choosing an A rather than a U. Again, these observations confirm the possibility of multiple binding registers. For AU15-bound RRM3, fitting of both A and U in position 4 was tested. After refinement, U fits the electron density better than A (**Supplementary Figure 3C**).

NMR-based analysis of HuR RRM3-RNA interactions

In the presence of U4, which is expected to be shorter than the optimal binding sequence (see above and **Supplementary Figure 2C**) much smaller NMR spectral changes are observed as compared with longer RNAs (**Supplementary Figure 4B and 5**). U9 represents a target sequence with multiple binding registers, and spectral changes upon addition of U9 are comparable with U6-induced changes. In contrast, the AU17 ligand leads to line broadening of residues close to the RNP1 pocket (*e.g.* Phe289) indicating co-occurrence of binding events with an A or U in this pocket (**Supplementary Figure 4B**).

RRM dimer interfaces from crystal structures of Trx-RRM3 and RNA-bound RRM3

In the Trx-RRM3 crystal, hydrogen bonds (Gln262 - Gly258) and Trp261 indole ring - Pro266 interactions are formed between α1 helices (**Supplementary Figure 6A**). In RRM3-RNA crystals, three interfaces can be seen between RRM3 molecules (within and between asymmetric units) (**Supplementary Figure 6B-D**). One of the interfaces (chain A and B) is formed between α1 helices but it has a different geometry than in Trx-RRM3 crystal. We observe stacking between Trp261 indole rings and hydrogen bonds between Gly265 and Val270 (loop between α1 and β2). Chain A and C have α2 helices in close proximity with hydrogen bonds between Tyr295 and Ser304. Finally, the helix α1 from chain C interacts with N- and C-terminal amino acids (Asp256 - Phe319; Gln262 - Lys320; Gln262 - Cys245). An additional hydrogen bond is found between Glu296 and Asp312. The divergent orientations of the interface in A as compared with B is probably induced by crystal packing in the Trx-RRM3 crystal.

SAXS analysis

Based on SEC-SAXS experiments, the molecular weight of unbound HuR W261E corresponds to 42 kDa, as calculated with SAXS MoW web tool (1) (**Supplementary Figure 6F**). This value is larger but within ±20% of the theoretical molecular weight of 37 kDa). The molecular weight derived from the POROD volume corresponds to 40 kDa. The slightly higher experimental value derived from POROD reflects the presence of a water shell around the protein and a flexible region.

The pairwise distance distribution P(*r*) of the unbound HuR W261E shows a shoulder, which indicates a certain separation between the domains, whereas the RNA bound form has a much steeper slope showing a more compact conformation of the complex. This is further supported by the decrease of D_{max} between free and bound HuR W261E (**Figure 5C**).

The Kratky plots reflect the folding state and the compactness of a protein. When comparing Kratky plots of free and RNA-bound HuR W261E, it can be appreciated that the peak of the bound form is sharper and the slope on the right side of the peak is steeper (**Supplementary Figure 10C**). This indicates that the RNA-bound form is more compact than the free form. The plot with free forms of HuR W261E and HuR GGS shows that these are quite similar; suggesting that in both constructs the linker is flexible and unstructured in a similar way (**Supplementary Figure 10E**).

To obtain a structural model of the full-length HuR protein we have employed the EOM analysis as described by Bernado et al (2) and Tria et al (3). The EOM software generates a pool of structures and selects the most probable ones together with their frequency of occurrence based on best fitting to the scattering curve. The frequency percentages are part of the ensemble selection process and not calculated separately.

As SAXS data indicate a homogenous globular domain arrangement of the HuR W261E/RNA complex, a structural model was created using Coral (4) based on the crystallographic structures of RRM1,2 and RRM3 in complex with their respective RNA ligands (4EGL and 6G2K). Based on the NMR data **(Figure 5A-B, Supplementary Figure 8, 9)** and on the quality of the fit to the experimental SAXS curves, we placed RRM3 upstream of the RRM1,2 binding site at the 5' end of the RNA and in close proximity to RRM2 (**Figure 5D, Supplementary Figure 10H**). Due to the significant length of the flexible linker connecting RRM2 and RRM3 it is physically possible that RRM3 could also bind at the 3' end downstream of RRM1,2. For the modeling, we tested both possibilities by enforcing distance constraints for the overall domain positions. These calculations showed that the arrangement of RRM3 at the 5' end to be of lowest energy (data not shown), although the alternative topology cannot be strictly excluded.

ARE motifs in 3'UTRs of mRNAs selected as cellular targets of HuR

To assess the contribution of RRM3 to the function of HuR in human cells, we selected previously described RNA targets of HuR, whose AREs in 3´UTRs are underlined according to literature references in brackets. For cyclins the available literature (5) does not specify the ARE, so individual ARE motifs were underlined. Most of these sites are also retrieved in HuR CLIP/PAR-CLIP datasets (6-8).

c-fos ARE (Peng et al, *ref* 9). Predominant binding sites according to *ref.* (10) are shown in bold.

ggggcagggaaggggaggcagccggcacccacaagtgccactgcccgagctggtgcattacagagaggagaaacacatcttccctag
agggttcctgtagacctagggaggaccttatctgtgcgtgaaacacaccaggctgtgggcctcaaggacttgaaagcatccatgtgtggactc
aagtccttacctcttccggagatgtagcaaaacgcatggagtgtgtattgttcccagtgacacttcagagagctggtagttagtagcatgttgagc
caggcctgggtctgtgtctcttttctctttctccttagtcttctcatagcattaactaatctattgggttcattattggaattaacctggtgctggatattttca
aattgtatctagtgcagctgattttaacaataactactgtgttcctggcaatagtgtgttctgattagaaatgaccaatattatactaagaaaagatac
gactttattttctggtagatagaaataaatagctatatccatgtactgtagtttttcttcaacatcaatgttcattgtaatgttactgatcatgcattgttgag
gtggtctgaatgttctgacattaacagttttccatgaaaacgttttattgtgtttttaatttatttattaagatggattctcag**atatttatatttttattttatttt**

tttctaccttgaggtcttttgacatgtggaaagtgaatttgaatgaaaaatttaagcattgtttgcttattgttccaagacattgtcaataaaagcattt
aagttgaatgcgaccaa

PTMA ARE (Lal et al, *ref* 11). Predominant binding sites according to *ref.* (12) are shown in bold.

acagcaaaaaaggaaaagttaaactaaaaaaaaaaaggccgccgtgacctattcaccctccacttcccgtctcagaatctaaacgtggtca
ccttcgagtagagaggcccgcccgcccaccgtgggcagtgccacccgcagatgacacgcgctctccaccacccaacccaaaccatgaga
atttgcaacaggggaggaaaaaagaaccaaaacttccaaggccctgcttttttcttaaaagtactttaaaaa**ggaaatttgtttgtattttt**tattt
acattttatattttgtacatattgttagggtcagccatttttaatgatctcggatgaccaaaccagccttcggagcgttctctgtcctacttctgactttac
ttgtggtgtgaccatgttcattataatctcaaaggagaaaaaaaaccttgtaaaaaaagcaaaaatgacaacagaaaaacaatcttattccga
gcattccagtaactttttgtgtatgtacttagctgtactataagtagttggtttgtatgagatggttaaaaaggccaaagataaaaggtttcttttttttc
ctttttgtctatgaagttgctgtttattttttttggcctgtttgatgtatgtgtgaaacaatgttgtccaacaataaacaggaattttattttgctgagttgttct
aaca

SIRT-1 ARE (Abdelmohsen et al, ref 13).

tgtaataattgtgcaggtacaggaattgttccaccagcattaggaactttagcatgtcaaaatgaatgtttacttgtgaactcgatagagcaagga
aaccagaaaggtgtaatatttataggttggtaaaatagattgtttttcatggataattttaacttcattatttctgtacttgtacaaactcaacactaact
ttttttttttaaaaaaaaaaaggtactaagtatcttcaatcagctgttggtcaagactaactttcttttaaaggttcatttgtatgataaattcatatgtgta
tatataattttttttgttttgtctagtgagtttcaacatttttaaagttttcaaaaagccatcggaatgttaaattaatgtaaagggaacagctaatctaga
ccaaagaatggtattttcacttttctttgtaacattgaatggtttgaagtactcaaaatctgttacgctaaacttttgattctttaacacaattatttttaaac
actggcattttccaaaactgtggcagctaactttttaaaatctcaaatgacatgcagtgtgagtagaaggaagtcaacaatatgtggggagagc
actcggttgtctttacttttaaaagtaatacttggtgctaagaatttcaggattattgtatttacgttcaaatgaagatggcttttgtacttcctgtggacat
gtagtaatgtctatattggctcataaaactaacctgaaaaacaaataaatgctttggaaatgtttcagttgctttagaaacattagtgcctgcctgga
tccccttagttttgaaatatttgccattgttgtttaaatacctatcactgtggtagagcttgcattgatcttttccacaagtattaaactgccaaaatgtga
atatgcaaagcctttctgaatctataataatggtacttctactggggagagtgtaatattttggactgctgttttccattaatgaggagagcaacagg
cccctgattatacagttccaaagtaataagatgttaattgtaattcagccagaaagtacatgtctcccattgggaggatttggtgttaaataccaaa
ctgctagccctagtattatggagatgaacatgatgatgtaacttgtaatagcagaatagttaatgaatgaaactagttcttataatttatctttatttaa
aagcttagcctgccttaaaactagagatcaactttctcagctgcaaaagcttctagtctttcaagaagttcatactttatgaaattgcacagtaagc
atttatttttcagaccatttttgaacatcactcctaaattaataaagtattcctctgttgctttagtatttattacaataaaaagggtttgaaatatagctgtt
ctttatgcataaaacacccagctaggaccattactgccagagaaaaaaatcgtattgaatggccatttccctacttataagatgtctcaatctgaa
tttatttggctacactaaagaatgcagtatatttagttttccatttgcatgatgtttgtgtgctatagatgatattttaaattgaaaagtttgttttaaattatttt
tacagtgaagactgttttcagctctttttatattgtacatagtcttttatgtaatttactggcatatgttttgtagactgtttaatgactggatatcttccttcaa
cttttgaaatacaaaaccagtgtttttttacttgtacactgttttaaagtctattaaaattgtcatttgactttttttctgttaactt

β-catenin ARE (Lopez de Silanes et al, ref 14)

atcatcctttagctgtattgtctgaacttgcattgtgattggcctgtagagttgctgagagggctcgaggggtgggctggtatctcagaaagtgcctg
acacactaaccaagctgagtttcctatgggaacaattgaagtaaactttttgttctggtccttttttggtcgaggagtaacaatacaaatggattttgg
gagtgactcaagaagtgaagaatgcacaagaatggatcacaagatggaatttagcaaaccctagccttgcttgttaaaattttttttttttttttttttaa
gaatatctgtaatggtactgactttgcttgctttgaagtagctcttttttttttttttttttttttttttgcagtaactgtttttttaagtctctcgtagtgttaagttatag
tgaatactgctacagcaatttctaatttttaagaattgagtaatggtgtagaacactaattaattcataatcactctaattaattgtaatctgaataaag
tgtaacaattgtgtagccttttttgtataaaatagacaaatagaaaatggtccaattagtttccttttttaatatgcttaaaataagcaggtggatctatttc
atgtttttgatcaaaaactatttgggatatgtatgggtagggtaaatcagtaagaggtgttatttggaaccttgttttggacagtttaccagttgcctttta
tcccaaagttgttgtaacctgctgtgatacgatgcttcaagagaaaatgcggttataaaaaaatggttcagaattaaacttttaattcatt

Cyclin A ARE (Wang et al, ref 5)

gtgaaagactgccggctttgtttgaaacaggagtcgctcggagtccatgctgtacagtttttatgtcgggttttagtttcacaatcacttctgaatgta
gatggtatagccacagacaaattacggtatccattgcttttaaaatggttttaatttgtatatcttttgtacatgtgtctatttggataattttaattggctaa
cattggtgctgccacctgtcaagatattgagaataaactgatttggaaatctttgcaagtcaaagttaatctgaggtaaaaatatgaatgatgtcat
ttagaaactaacttgatttgttttattgtacagtggggagtaggaaaagattttataaaactaaaatatttcagggaaaaagaccttcaaattctggc
attgattgattattctaagcaaccaaaccaaattgctgacttattcataaataaccaaaagtagacaaatgtgttttcaatgtagcagaactcattc
ggctctcacatgttacagtagtttatggagacattttatttatcaacttttacaaataaggtcttacatgagttgggaagcttgtgcaacagaaaagttt

tccagaaagtttattcaaagcataatgttaaagacaaaacagtgtatcaaggagttcagcttgtaagatatccagagctgttagcactgagtatgt
atacacacacacacacacacatacacacacacacagatacaccactttagcttagttagcagttctcttcttcttccttttcccttggcctttacttgg
tgccttctggtttgcactggtctgagctctgtgaatgaagtaatataacagctcactgtatcccatctgtcctggattgggtcactgggactctgaaa
ggtgccccattttatagaaccagcttttaatgtatatttgaaagaggccagaaatttgttaccttattttctttgggttcttctctggctccaaaataatcat
ttcttcttcttcactatctgatagcactatagggg caccttt ggaaaagtccaaaaaaggagtttactgcttaagcacatcagagtcctaacgctcc
catctccaggctaagggagctcccaagctctactgcccggaggctgacactctttccgaatcttaccctgttctaagtaagtagctccttgcagct
ctgaaatttgtaaaaattgatgttttctataaactctttctttcctttttccccagaggacatgtaggccaggatgctctaattcttgctaccaggactg
cccatgaaaaatgtagaaaactgctttgtttgcccatttttctcttgaacagtaattatatatgtgtgtaaataaagcatgcaaattaaccctttaaaa
aaaaaaaaaaaaa

Cyclin B1 ARE (Wang et al, ref 5)

Tccaatagactgctacatctgcagatgcagttagccatgtgccgcctgtacataggatacctaccgtgtttacttgctcttcaataaaggttgtgac
ttctcattttacatagcttaactcatttgaatgttgttgcttctgagtttaggctaacggaagttgtcgaatttaggagtatattaaaaactgcatctagtttt
aacagtggatccaactaatgtatatatctgtagcctatatgtctatatacatccttcactgtgtgtccttatatcatcatgtcttctgcctcactctagttta
aactctaaatctaccagctagtcctttgttccattttccagtggttgccacctttaaccactgtctcttggttggtcaactttcagatctgaaaccaagta
tcttttttatgtaattatttatttgttcttaattggaaaataggatgttcaaaattaaaggtgtgttttaaaaagaatttgcccccaagtctcactatcaac
agataagggtgtatcttgtatatcctgtatagaaataatcatgcatatactcccaaggagatattttatatgggttcatttcatcaacagtattcctatc
agcattccttacaatgcctatattgcatctcctagtgtgaacaaactgtgtgtaacatagtcattccctcgggggggattcaagtgcattctctcagt
gccctccacagtgttcttaaatgatgtttaatgtcttgcttggctcattcatagtagtcttccagggggtgctttgaattctgacagccagatgggtgtg
gctgccaccataccaaggcgccactcctgtcttgtaatgccacctggaaaagaatcctgtctcatttgctgttttaatttatacatctgatatcaagtt
gaataaaatttattggtggaaagctttcacaatt

Supplementary Methods

Spin labelling

N- and C-terminally truncated HuR (18-323) (RRM1-3) was expressed as described for full-length HuR. The N-terminal truncation eliminated the single cysteine residue present outside of RRM3. RRM3 contains two cysteines, of which only one is surface-exposed (Cys245) based on the three-dimensional structure. The purified protein was dialyzed to DTT-free buffer and divided into two portions, of which one was incubated with 10x molar excess of IPSL. We thus obtained two HuR samples, with and without the spin label. After labelling, the two HuR samples were dialyzed to NMR buffer without DTT and the NMR spectra were recorded. Reduction of the spin-labelled protein sample with ascorbic acid could not be conducted, as HuR NMR sample could not be subjected to two subsequent spectra acquisitions (10 hours each) due to its limited stability at room temperature.

qPCR Supplemental information

Experimental design

In the analysis of the HuR mRNA target levels and the ribonucleoprotein formation, the control group was composed by the HuR WT-overexpressing RKO cells, whereas the experimental groups were the different mutated HuR-overexpressing RKO cells. Each group was composed of three independent experimental samples.

Sample

Each sample was composed of RKO cells plated in one well of a six-well plate. At the end of the experiment, cells were immediately lysed with Trizol reagent (Invitrogen) and snap frozen. The Trizol-lysed extracts were stored at -80ºC until RNA extraction. In the case of the samples for the RNP-IP, the RNA was immediately extracted after the end of the experiment as described in the corresponding section.

RNA isolation

RNA was isolated from cell lines using Trizol (Invitrogen) according to manufacturer's instructions or by the described RNP-IP protocol. RNA concentration was determined spectrophotometricallyusing Nanodrop ND-1000 Spectrophotometer (Thermo Scientific). 1 µg of the obtained RNA was treated with DNase I (Invitrogen) according to manufacturer's protocol. RNA integrity was checked by electrophoresis in a 1% agarose gel with ethidium bromide for visualization. Inhibition tests waere not performed.

Reverse transcription and Real Time quantitative PCR (RT-qPCR)

cDNA was synthesized with M-MLV (Invitrogen) by using the DNAse I-treated RNA in the presence of random primers and RNaseOUT (Invitrogen), according to manufacturer´s instructions. Resulting cDNA was stored at -20ºC until subsequent analysis.

cDNA was diluted 1/10 in RNase free water (Sigma-Aldrich), and 1,5 µL were used for qPCR reaction. qPCRs were performed using ViiA7 thermocycler (Applied Biosystems) with SYBR Select (Applied Biosystems) and specific primers in a total reaction volume of 6 µl. All reactions were performed in triplicates following manufacturer's conditions. Primers were designed using PrimerBlast online resource and synthetized by Sigma-Aldrich (desalted and without modifications). The no-RT control showed no amplification Cq.

The specificity of the PCR reaction was controlled by monitoring the melting curves. The mRNA fold abundance was calculated by the ΔΔCt method, using GAPDH as reference gene with the QuantStudio program (Applied Biosystems) and Excel 2016. The statistical significance was determined using two-tail Student's t ($p<0.05$) in Excel 2016.

The oligonucleotides are listed in the Table below.

**Target mRNA**	**Forward primer**	**Reverse primer**
Cyclin E	ACAATAATGCAGTCTGTGCA	ATAGACTTCACACACCTCCA
Cyclin A	CGGTACTGAAGTCCGGGAAC	GTGACATGCTCATCATTTACAGGAA
Cyclin B1	CCTGATGGAACTAACTATGTTG	CATGTGCTTTGTAAGTCCTTGA
SMAD-2	GCCATCACCACTCAAAACTGT	GCCTGTTGTATCCCACTGATCTA
c-Fos	CAGACTACGAGGCGTCATCC	TCTGCGGGTGAGTGGTAGTA
SIRT-1	GAGGAGGCGAGGGAGGAG	GCTGAAGGGCGAGGGC
β-CATENIN	TTGAAGGTTGTACCGGAGCC	GCAGCTGCACAAACAATGGA
PTMA	CAGCTTTATCGCCAGAGTCC	AGTCCTTGGTGGTGATTTCG
GAPDH	AATGAAGGGGTCATTGATGG	AAGGTGAAGGTCGGAGTCAA

Supplementary Figures

Supplementary Figure 1

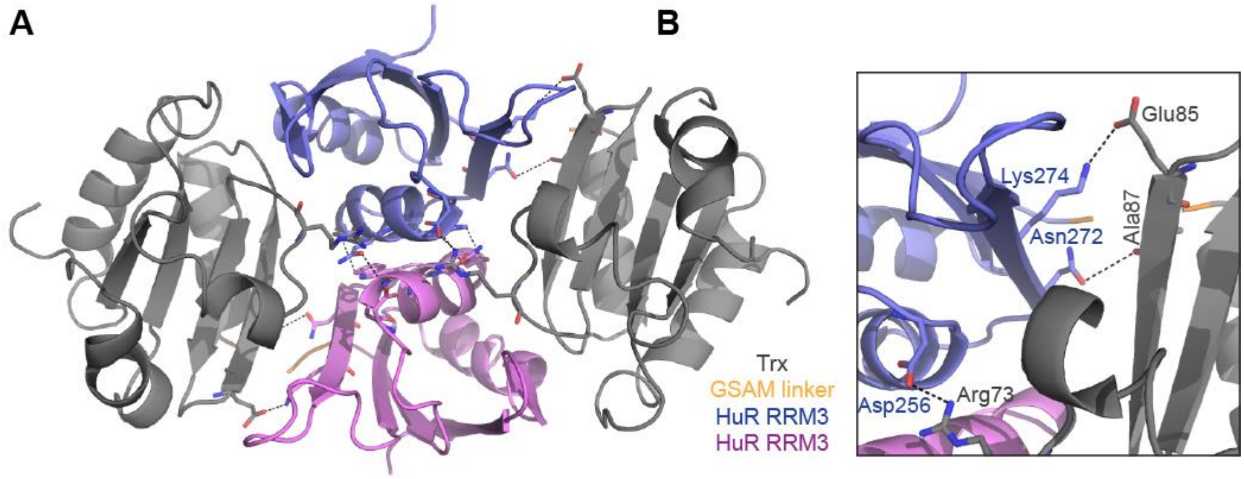

Arrangement of Trx-RRM3 molecules in the asymmetric unit.

A. Carton representation of Trx-RRM3 molecules in the asymmetric unit. Residues forming intermolecular hydrogen bonds are marked as sticks. Hydrogen bonds are marked as dashed lines.

B. Zoomed view showing hydrogen bonds between RRM3 and Trx.

Supplementary Figure 2

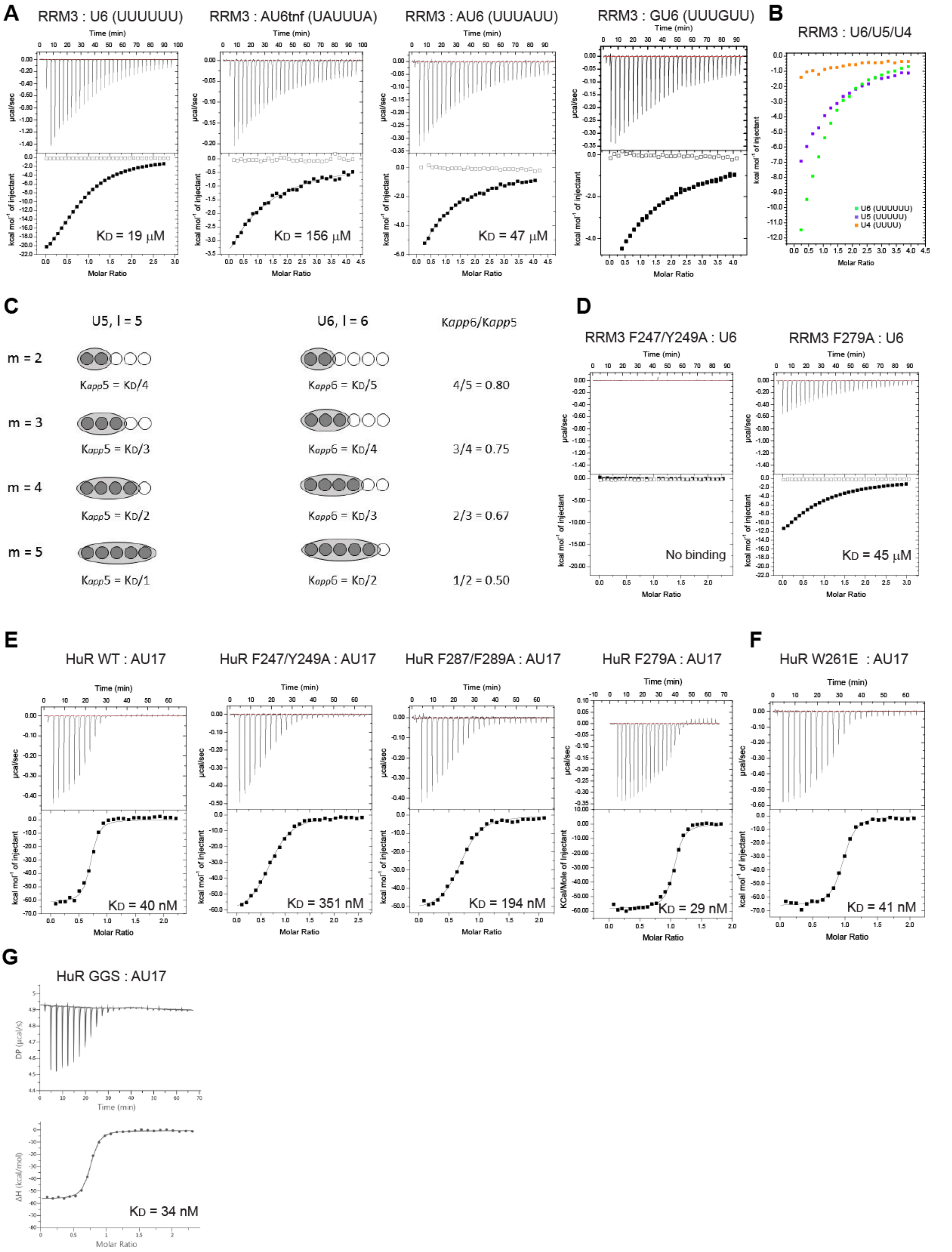

Isothermal titration calorimetry of HuR constructs and RNAs

A. 600-900 μM RNAs were titrated to 40-50 μM RRM3. The graphs are representative of 2-3 independent ITC measurements.

B. Overlay of raw ITC data for U4, U5 and U6 RNAs (400 µM) titrated to RRM3 (20 µM).

C. Estimation of the length of RRM3 minimal binding site on U-rich RNA ligand using the model of multiple binding registers (15). Different length ligands are presented as circles, binding sites as dark grey circles and RRM3 domain as light grey ovals. The K_d values were calculated based on ITC experiments where 400 µM U6 and U5 RNA were titrated to 20 µM of RRM3.

D. 400-600 µM U6 RNA was titrated to 20-40 µM solutions of RRM3 mutants. The graphs are representative of two independent ITC measurements.

E-G. 60 µM AU17 RNA was titrated to 5 µM HuR WT and mutants. The graphs are representative of 2-3 independent ITC measurements.

A, D-G. Control titrations of RNA to the buffer were subtracted from experimental runs before K_d calculations.

Supplementary Figure 3

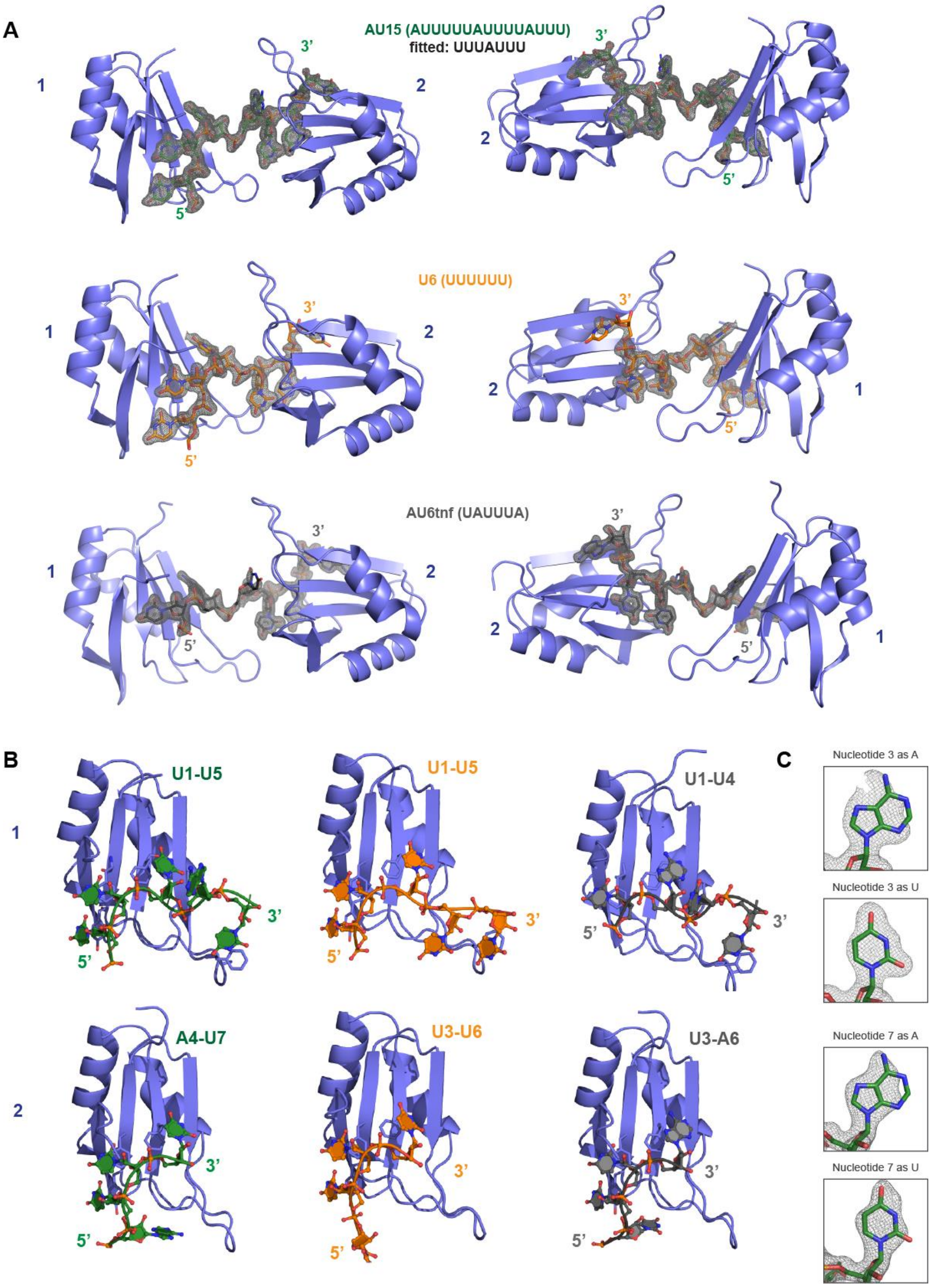

Arrangement of macromolecules in the crystals of RNA-bound RRM3

A. View of RRM3 molecules (cartoon) with RNA ligands indicated as sticks and with a 2Fo-Fc electron density map contoured at 1.2σ. In case of RRM3-AU15, 7 nucleotides could be fitted into the electron density (UUUAUUU).

B. Cartoon representations of individual RRM3 chains with adjacent nucleotides from RRM3-RNA crystals. The RNA ligands are presented using the following color-code:AU15 - green, U6 - orange and AU6tnf - grey.

C. Fitting of nucleotide in position 4 in the model of RRM3 bound to AU15. Electron density map of the nucleotide stacked with Phe289 from RNP1 fitted as an A or as a U. U appears to be the best fit.

Supplementary Figure 4

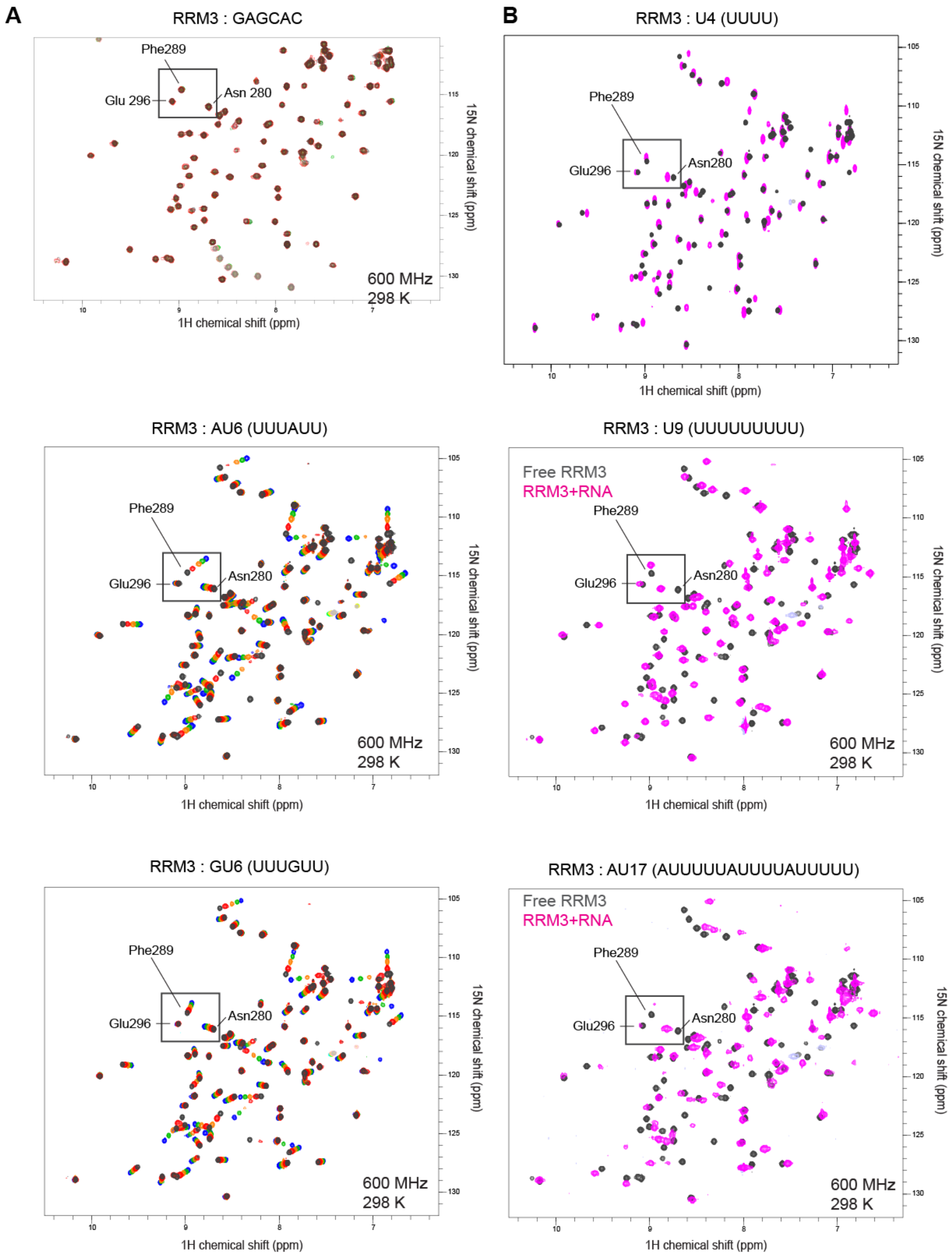

HSQC NMR spectra of HuR RRM3 titrated with RNAs

A. $^{1}H,^{15}N$-HSQC NMR spectra of 120 µM HuR RRM3 free (black) and titrated with increasing amounts of the RNA ligands -GAGCAC, AU6 and GU6. Two titration points were recorded for GAGCAC (1:0.25 and 1:1.25). For AU6 and GU6 RNAs, four titration points were recorded (from 1:0.25 to 1:4.5).

B. $^{1}H,^{15}N$-HSQC NMR spectra of 120 µM HuR RRM3 free (black) in the presence of 1.25 molar excess of U4, U9 or AU17 (magenta).

A and B. Asn280, Phe289 (exhibit different chemical shift changes depending on the presence of A or U containing RNAs) and Glu296 are highlighted by a box.

Supplementary Figure 5

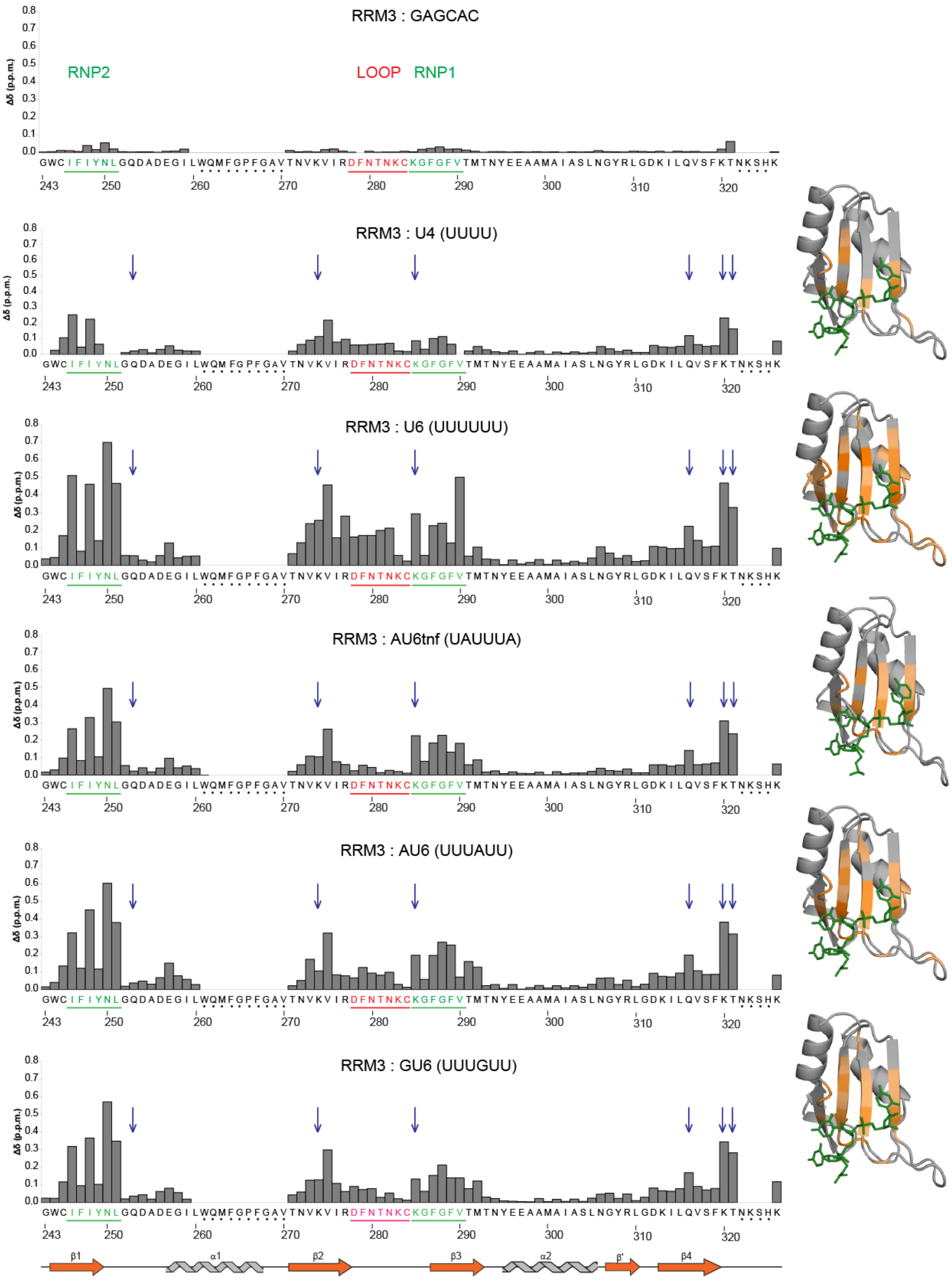

Graphical representation of CSPs induced by titration of RNAs to HuR RRM3

CSPs in the presence of 1.25 molar excess of RNAs. Black dots indicate residues where amide signals were undetectable in RRM3 spectra. Secondary structure elements of HuR RRM3 are depicted below the graphs. RNA-binding areas observed in the crystal structures are marked in green (RNPs) and red (β2β3 loop). Blue arrows indicate residues involved in RNA recognition based on crystal structures. CSPs are plotted on the structure of RRM3 bound to core nucleotide sequence (nucleotides 2-4).

Supplementary Figure 6

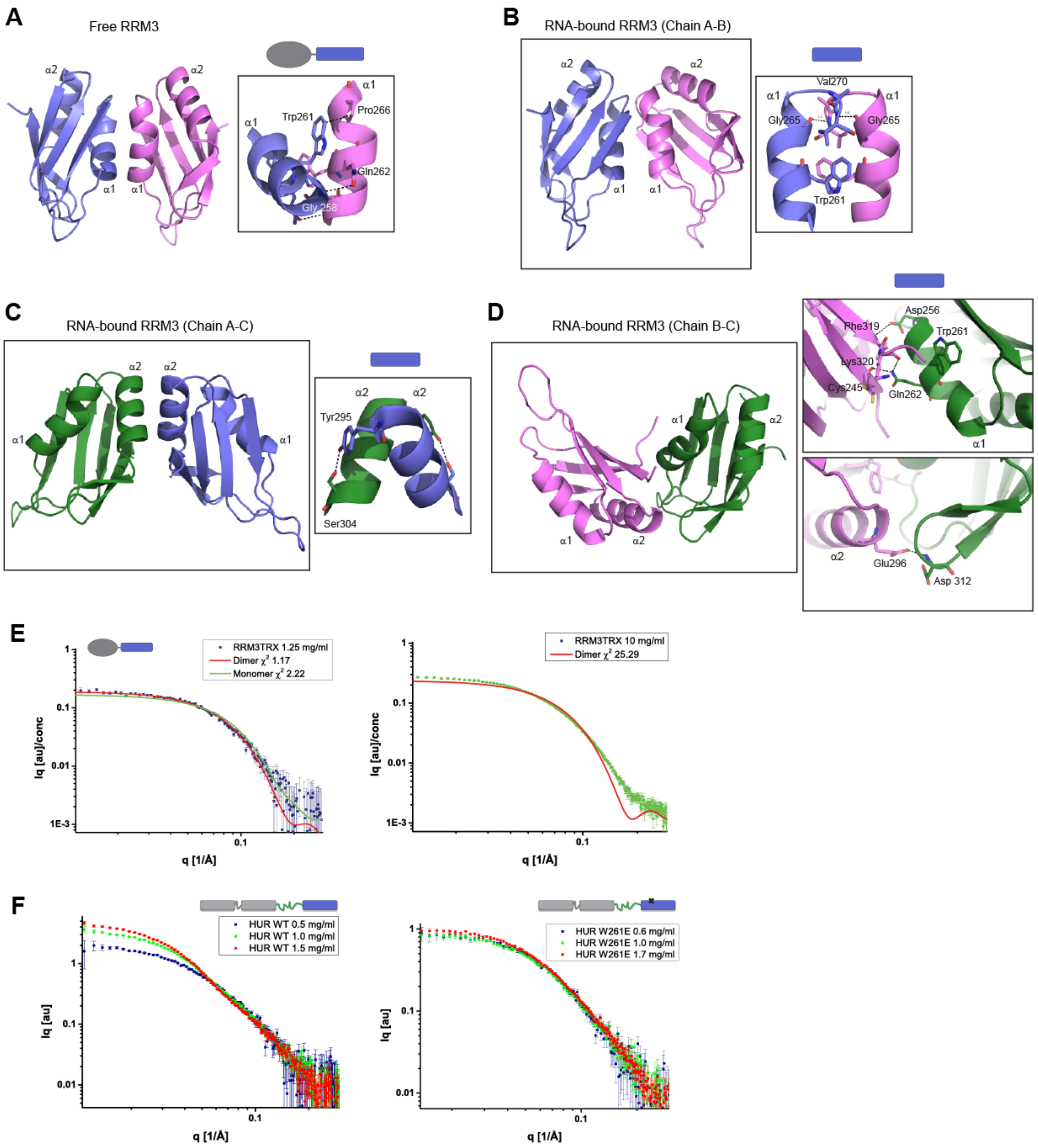

Estimated molecular weight

HuR WT	0.5 mg/ml	104.5 kDa
HuR WT	1.0 mg/ml	217.4 kDa
HuR WT	1.5 mg/ml	319.3 kDa

Estimated molecular weight

HuR W261E	0.6 mg/ml	42.0 kDa
HuR W261E	1.0 mg/ml	40.0 kDa
HuR W261E	1.7 mg/ml	44.9 kDa

HuR dimerization through RRM3

A. Cartoon representation of adjacent RRM3 molecules in the crystal structure of Trx-RRM3 and close-up on the interface. The zoomed view shows residues that stabilize the interface as sticks. Hydrogen bonds are indicated as dashed lines.

B-D. Cartoon representations (left) and zoomed views of the dimer interfaces (right) of adjacent RRM3 chains in crystal structures of RRM3-RNA complexes (both within and between asymmetric units). The structures shown correspond to the RRM3-U6 crystal, but are representative for the two other RRM3-RNA crystals.

E. Fitting of dimeric and monomeric forms of Trx-RRM3 to experimental SAXS data of Trx-RRM3 at lowest and highest tested concentration (1.25 and 10 mg/ml, respectively), with χ^2 values indicated.

F. Overlays of experimental SAXS data at increasing concentrations of HuR WT and HuR W261E. Molecular weights estimated from SAXS data are indicated below.

Supplementary Figure 7

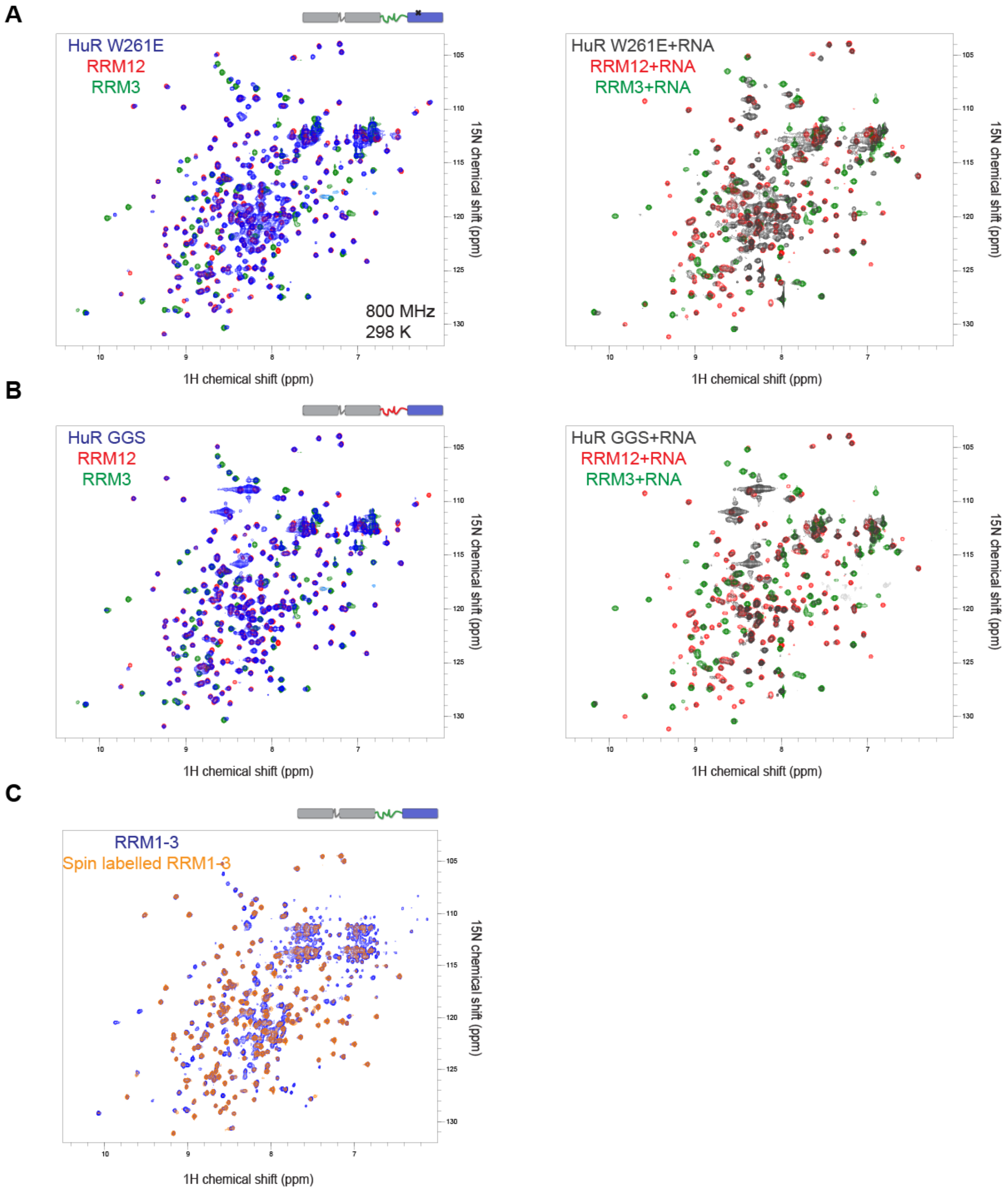

NMR experiments addressing the domain arrangement of HuR FL

A. Superpositions of ^{1}H,^{15}N correlation NMR spectra of free and RNA-bound HuR W261E (40 µM) with spectra of free and RNA bound RRM1,2 (RNA: AU12) and RRM3 (RNA: U6).

B. Superpositions of ^{1}H,^{15}N correlation NMR spectra of free and RNA-bound HuR GGS (70 µM) with NMR spectra of free and RNA-bound RRM1,2 (RNA: AU12) and RRM3 (RNA: U6).

C. Superposition of ^{1}H,^{15}N TROSY-HSQC spectra of unmodified deuterated RRM1-3 with deuterated RRM1-3 spin-labeled with IPSL at RRM3 Cys245.

Supplementary Figure 8

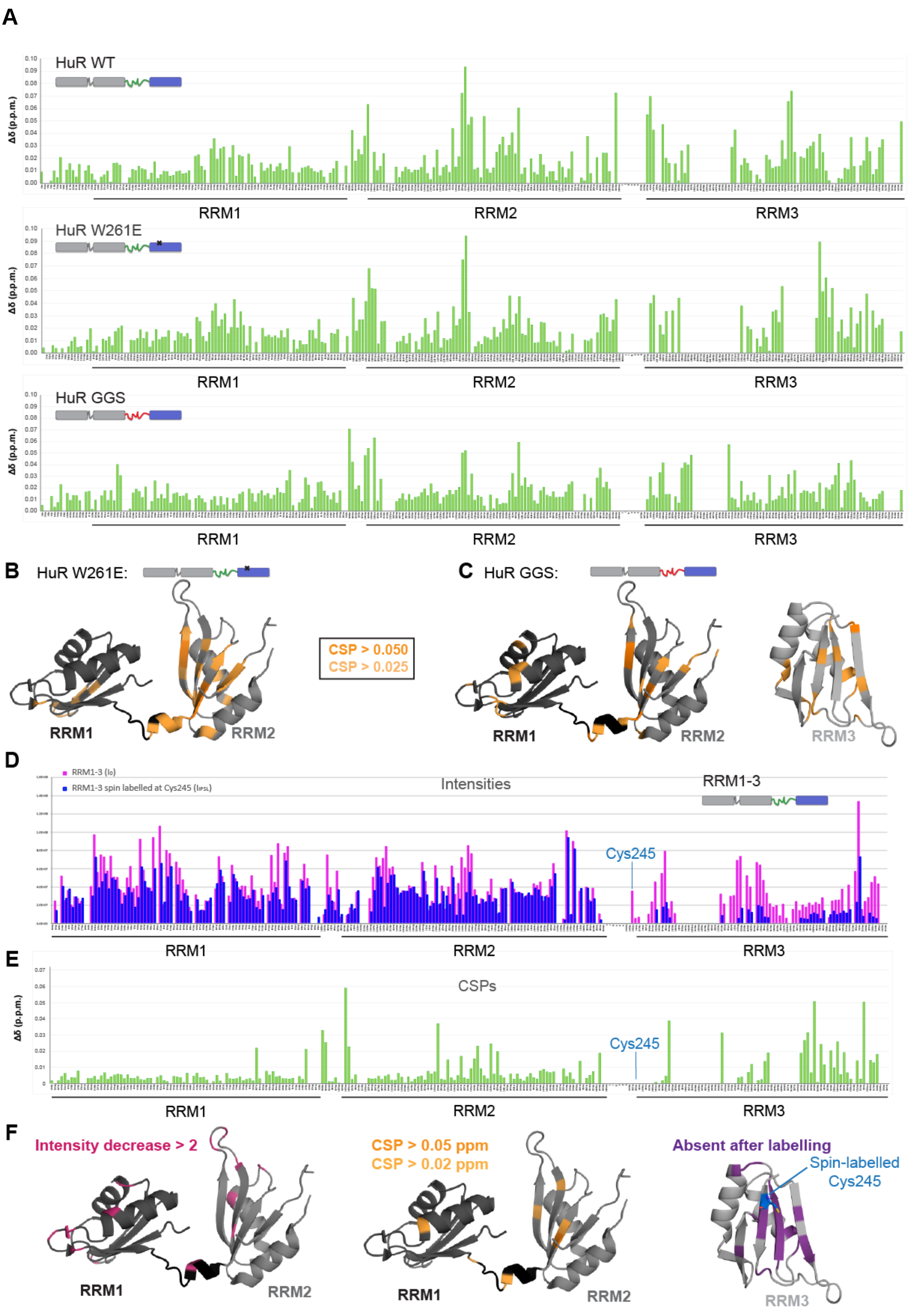

Analysis of interdomain contacts within free HuR based on NMR experiments

A. CSPs from comparing spectra of free full-length HuR variants (^{1}H,^{15}N TROSY-HSQC of deuterated HuR WT and ^{1}H,^{15}N HSQCs of HuR W261E and HuR GGS) with ^{1}H,^{15}N HSQC spectra of RRM1,2 and RRM3. The spectra of deuterated HuR WT were compared to spectra of deuterated RRM1,2 and RRM3 (TROSY vs HSQC shifts were corrected before CSP analysis).

B. CSPs of HuR W261E compared to RRM1,2 are plotted on the RRM1,2 structure. Mutation of Trp261 to Glu causes significant changes of RRM3 amide signals, which were not assigned. Therefore, CSPs were not plotted onto the RRM3 structure (not shown) for HuR W261E.

C. CSPs of HuR GGS compared to RRM1,2 and RRM3 are plotted on RRM1,2 and RRM3 structures.

D-F. PREs measured with deuterated RRM1-3 spin-labeled at RRM3 Cys245.

D. Intensities from ^{1}H,^{15}N TROSY-HSQCs of spin-labelled and unlabeled RRM1-3 (20 μM) versus HuR residue number. **E.** CSPs comparing spectra described in **(D)**.

F. Intensity changes and CSP from **(D-E)** are plotted on the structures of free RRM1,2 and RRM3.

Supplementary Figure 9

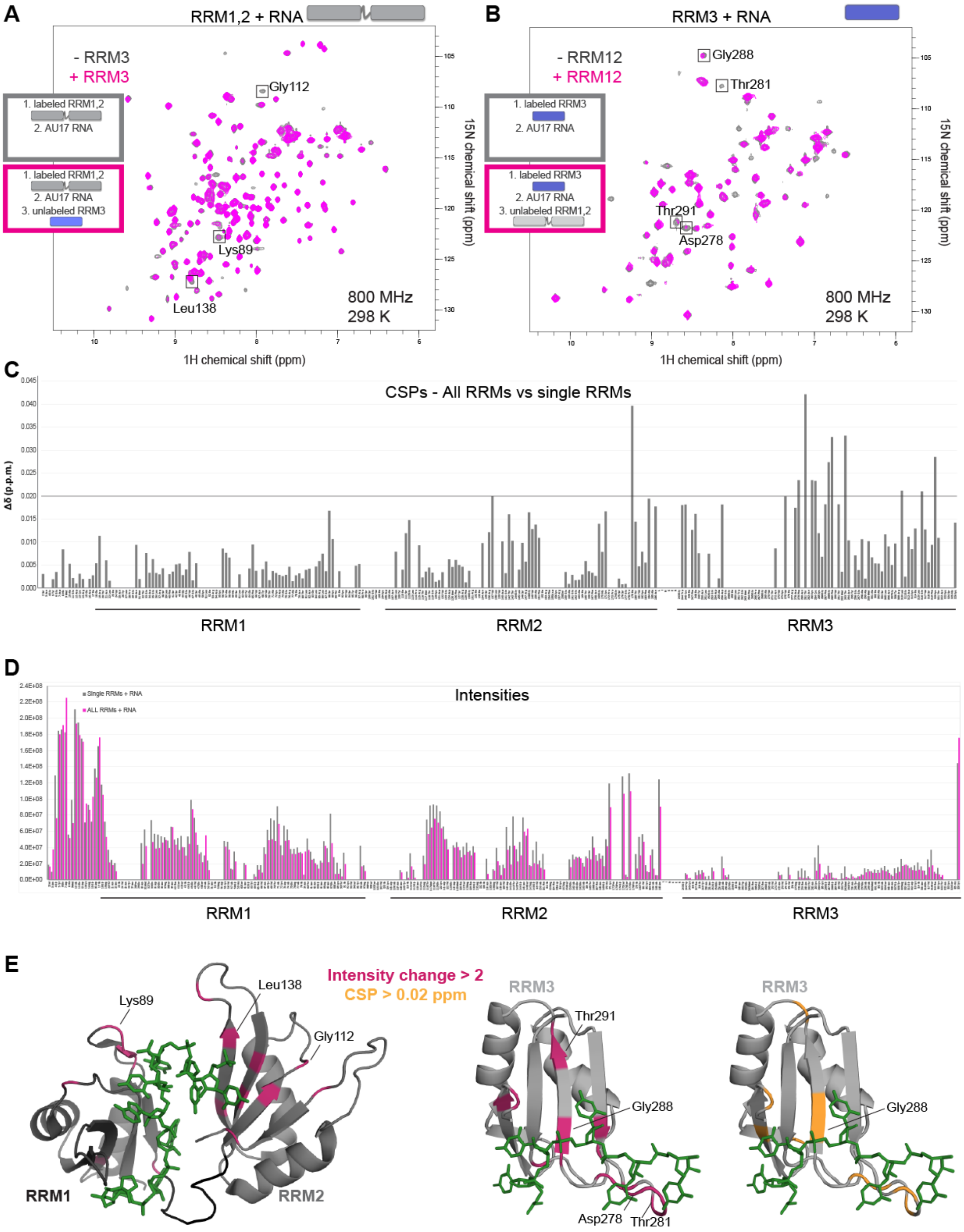

Analysis of interdomain contacts between RNA-bound RRM1,2 and RRM3

A. Overlay of $^{1}H,^{15}N$-HSQC spectra of $^{2}H,^{15}N$-labeled RRM1,2 + AU17 RNA (grey) and $^{2}H,^{15}N$-labeled RRM1,2 + AU17 + unlabeled RRM3 (magenta).

B. Overlay of $^{2}H,^{15}N$-labeled RRM3 + AU17 (grey) and $^{2}H,^{15}N$-labeled RRM3 + AU17 + unlabeled RRM1,2 (magenta).

A-B. Only the ^{15}N-labeled proteins are detected in the NMR spectra, thus reducing spectral complexity and signal overlap. The final concentrations of proteins and of the RNA were equal to 90 μM. Amide signals undergoing spectral changes (line broadening and/or CSPs) are highlighted by boxes and

annotated. **C.** CSPs were calculated comparing RRM1,2 and RRM3 bound to RNA with RRM1,2 and RRM3 bound to RNA in the presence of unlabeled RRM3 and RRM1,2, respectively. CSPs are plotted versus residue number in the upper graph.

D. Intensities of RRM1,2 and RRM3 bound to RNA and of RRM1,2 and RRM3 bound to RNA in the presence of unlabeled RRM3 and RRM1,2, respectively. The intensities are plotted versus residue number.

E. CSPs and intensity changes shown in **B-C** are plotted on the structures of RNA-bound RRM1,2 (PDB ID: 4ED5) and RRM3. Residues corresponding to peaks marked in **A-B** are localized on the structures of RRM1,2 and RRM3.

Supplementary Figure 10

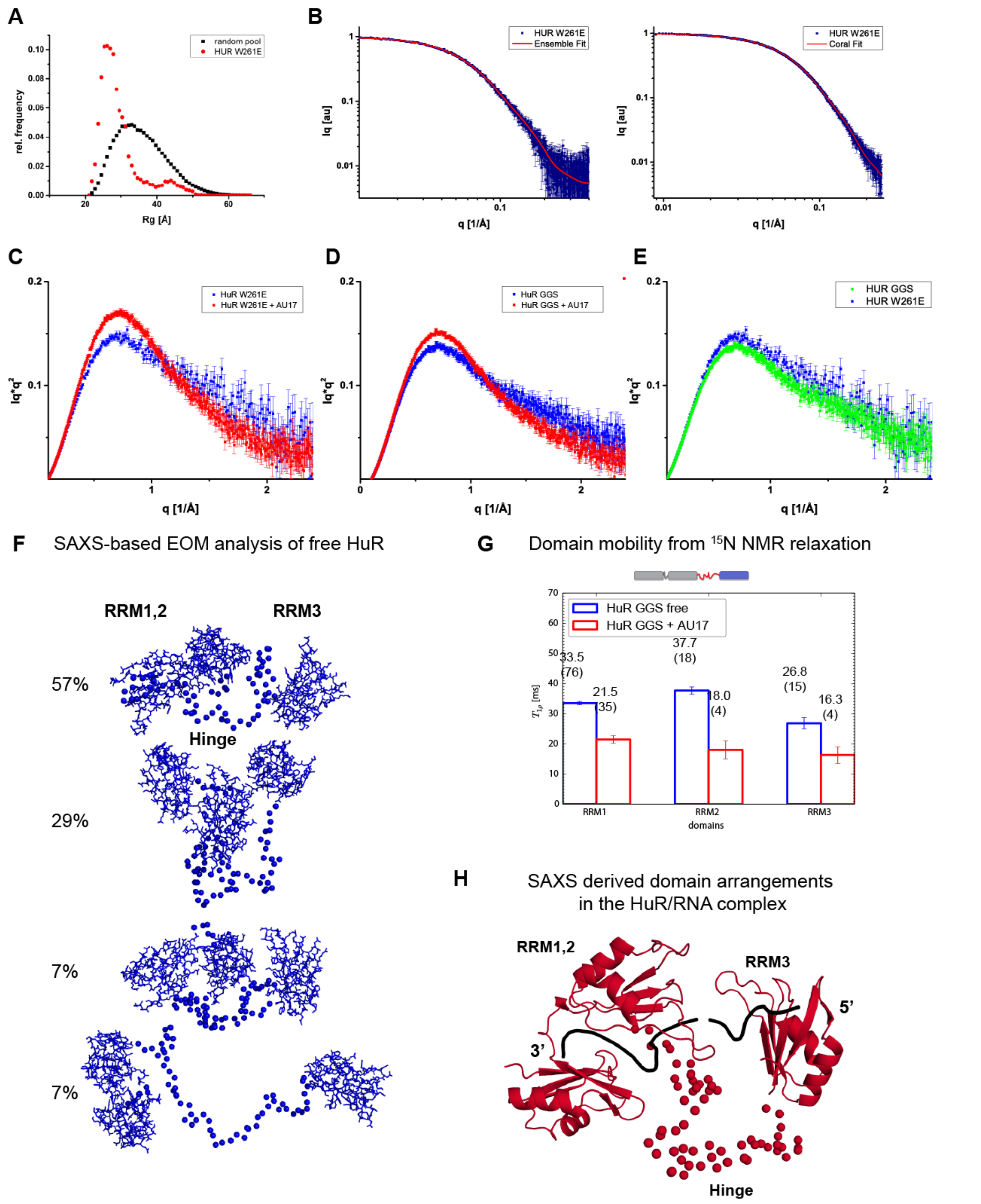

Supplementary Figure 10 – SEC-SAXS and NMR analysis of HuR free and RNA bound

A. Ensemble optimization analysis for HuR W261E: *R*g distribution for the pool of 10 000 conformers (black) and the selected best fitting structures (red) is shown.

B. Fits of SAXS data calculated for the structural models of free and RNA-bound HuR (red) superimposed with the experimental SAXS data (blue).

C-D. Superposition of Kratky plots of free and RNA-bound **(C)** HuR W261E and **(D)** HuR GGS. The Kratky plot shows the folding state and the compactness of a protein. The sharper peak and steeper slope at higher q-values seen for the RNA-bound proteins indicates that they are more compact than in the free state.

E. Overlays of Kratky plots of free HuR W261E and HuR GGS. The similar shape suggests that the GGS linker is comparable with the WT linker, *i.e*. it is unstructured and flexible.

F. Representative structural models of free HuR W261E resulting from the analysis of SAXS data using the ensemble optimization method (χ^2 = 0.82). The frequency percentage of the structural models is indicated. RRMs are shown as stick models (RRM1,2 on the left, RRM3 on the right), the RRM2-3 hinge linker is shown by blue spheres.

G. NMR ^{15}N $T_{1\rho}$ relaxation times are shown for HuR GGS in the absence (blue) and presence (red) of AU17 RNA. To allow for comparison of the domain dynamics, domain averaged relaxation times are shown for RRM1,2 and RRM3. The value in parentheses is the number of residues averaged.

H. Structural model of AU17-bound HuR W261E based on CORAL analysis (χ^2 = 0.83).

SAXS experiments shown in were coupled with size exclusion separation of the analyzed samples.

Supplementary Figure 11

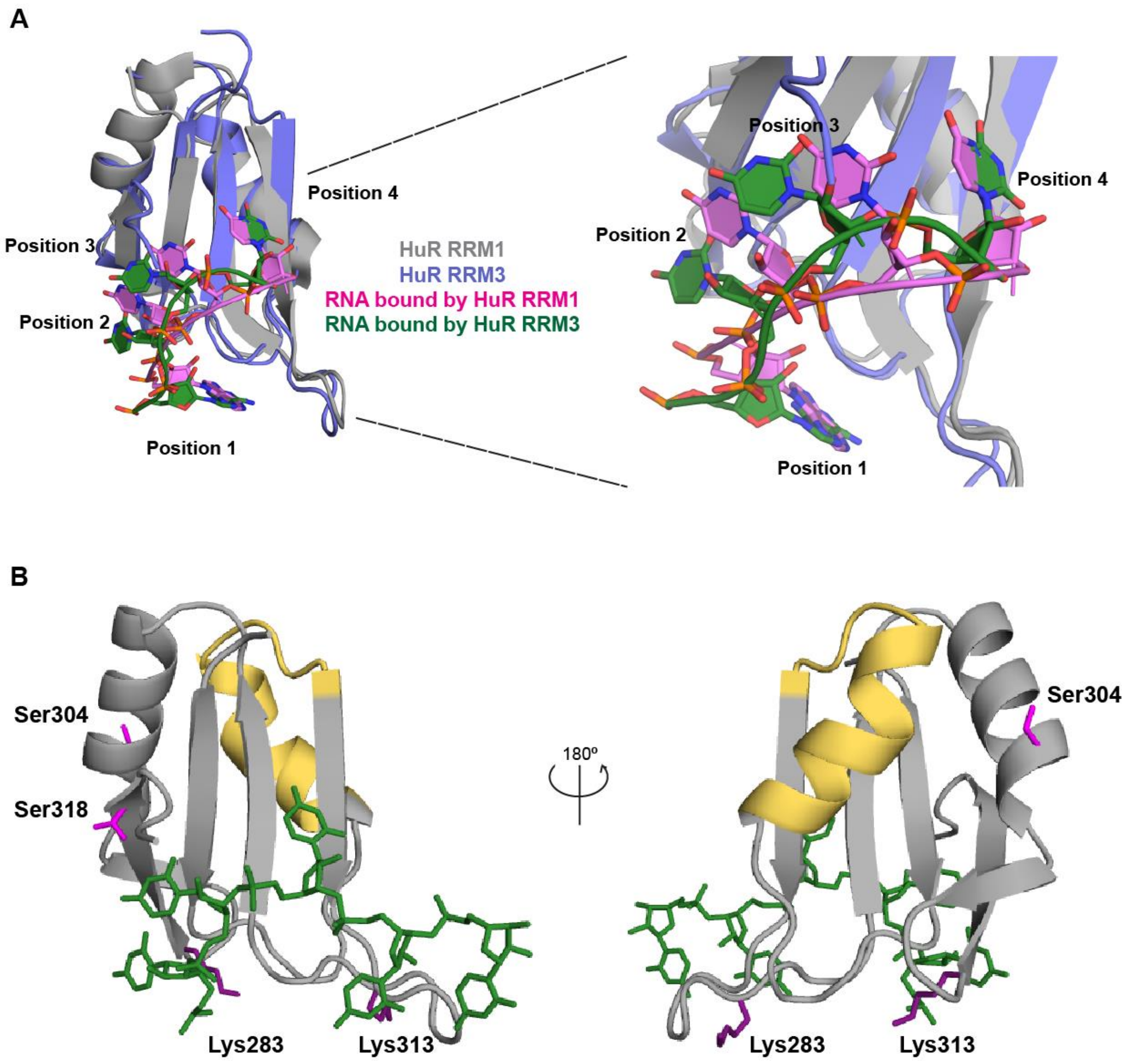

Supplementary Figure 11

A. Structural superposition of HuR RRM1-RNA (PDB ID: 4ED5) and RRM3-RNA (RRM3-AU15) complexes. RRMs are represented as cartoon, RRM1 in grey and RRM3 in blue. RNAs are shown as sticks with filled rings and colored according to atom type (oxygen - red, nitrogen - blue, phosphorus - orange). The RNA (AUUU) bound by RRM1 is shown in violet and by RRM3 in green.

B. Localization of postranslationally modified residues (presented as sticks) on the structure of RRM3. The RNA is shown as green sticks and the main dimerization interface is colored in yellow.

Supplemental References

1. Fischer, H., de Oliveira Neto, M., Napolitano, H.B., Polikarpov, I. and Craievich, A.F. (2010) Determination of the molecular weight of proteins in solution from a single small-angle X-ray scattering measurement on a relative scale. *Journal of applied crystallography*, **43**, 101-109.
2. Bernado, P., Mylonas, E., Petoukhov, M.V., Blackledge, M. and Svergun, D.I. (2007) Structural characterization of flexible proteins using small-angle X-ray scattering. *J Am Chem Soc*, **129**, 5656-5664.
3. Tria, G., Mertens, H.D., Kachala, M. and Svergun, D.I. (2015) Advanced ensemble modelling of flexible macromolecules using X-ray solution scattering. *IUCrJ*, **2**, 207-217.
4. Petoukhov, M.V., Franke, D., Shkumatov, A.V., Tria, G., Kikhney, A.G., Gajda, M., Gorba, C., Mertens, H.D., Konarev, P.V. and Svergun, D.I. (2012) New developments in the ATSAS program package for small-angle scattering data analysis. *Journal of applied crystallography*, **45**, 342-350.
5. Wang, W., Caldwell, M.C., Lin, S., Furneaux, H. and Gorospe, M. (2000) HuR regulates cyclin A and cyclin B1 mRNA stability during cell proliferation. *The EMBO journal*, **19**, 2340-2350.
6. Lebedeva, S., Jens, M., Theil, K., Schwanhausser, B., Selbach, M., Landthaler, M. and Rajewsky, N. (2011) Transcriptome-wide analysis of regulatory interactions of the RNA-binding protein HuR. *Molecular cell*, **43**, 340-352.
7. Mukherjee, N., Corcoran, D.L., Nusbaum, J.D., Reid, D.W., Georgiev, S., Hafner, M., Ascano, M., Jr., Tuschl, T., Ohler, U. and Keene, J.D. (2011) Integrative regulatory mapping indicates that the RNA-binding protein HuR couples pre-mRNA processing and mRNA stability. *Molecular cell*, **43**, 327-339.
8. Kishore, S., Jaskiewicz, L., Burger, L., Hausser, J., Khorshid, M. and Zavolan, M. (2011) A quantitative analysis of CLIP methods for identifying binding sites of RNA-binding proteins. *Nature methods*, **8**, 559-564.
9. Peng, S.S., Chen, C.Y. and Shyu, A.B. (1996) Functional characterization of a non-AUUUA AU-rich element from the c-jun proto-oncogene mRNA: evidence for a novel class of AU-rich elements. *Molecular and cellular biology*, **16**, 1490-1499.
10. Ma, W.J., Cheng, S., Campbell, C., Wright, A. and Furneaux, H. (1996) Cloning and characterization of HuR, a ubiquitously expressed Elav-like protein. *The Journal of biological chemistry*, **271**, 8144-8151.
11. Lal, A., Kawai, T., Yang, X., Mazan-Mamczarz, K. and Gorospe, M. (2005) Antiapoptotic function of RNA-binding protein HuR effected through prothymosin alpha. *The EMBO journal*, **24**, 1852-1862.
12. Lopez de Silanes, I., Zhan, M., Lal, A., Yang, X. and Gorospe, M. (2004) Identification of a target RNA motif for RNA-binding protein HuR. *Proceedings of the National Academy of Sciences of the United States of America*, **101**, 2987-2992.
13. Abdelmohsen, K., Pullmann, R., Jr., Lal, A., Kim, H.H., Galban, S., Yang, X., Blethrow, J.D., Walker, M., Shubert, J., Gillespie, D.A. *et al.* (2007) Phosphorylation of HuR by Chk2 regulates SIRT1 expression. *Molecular cell*, **25**, 543-557.
14. Lopez de Silanes, I., Fan, J., Yang, X., Zonderman, A.B., Potapova, O., Pizer, E.S. and Gorospe, M. (2003) Role of the RNA-binding protein HuR in colon carcinogenesis. *Oncogene*, **22**, 7146-7154.
15. Kelly, R.C., Jensen, D.E. and von Hippel, P.H. (1976) DNA "melting" proteins. IV. Fluorescence measurements of binding parameters for bacteriophage T4 gene 32-protein to mono-, oligo-, and polynucleotides. *The Journal of biological chemistry*, **251**, 7240-7250.
